# Examination of Elective Bariatric Surgery Rates Before and After US Affordable Care Act Medicaid Expansion

**DOI:** 10.1001/jamahealthforum.2021.3083

**Published:** 2021-10-08

**Authors:** Amresh D. Hanchate, Danyang Qi, Michael K. Paasche-Orlow, Karen E. Lasser, Zhixiu Liu, Mengyun Lin, Kristina Henderson Lewis

**Affiliations:** 1 Division of Public Health Sciences, Department of Social Sciences and Health Policy, Wake Forest School of Medicine, Winston-Salem, North Carolina; 2 Section of General Internal Medicine, Boston University School of Medicine, Boston, Massachusetts; 3 SuperMap International, Beijing, China; 4 Department of General Internal Medicine, Boston Medical Center, Boston, Massachusetts; 5 Department of Epidemiology and Prevention, Division of Public Health Sciences, Wake Forest School of Medicine, Winston-Salem, North Carolina

## Abstract

**Importance:**

There is limited evidence on whether the Affordable Care Act Medicaid expansion beginning in 2014 improved access to elective procedures. Uninsured individuals are at higher risk of obesity and may have experienced improved uptake of bariatric surgery following Medicaid expansion.

**Objective:**

To examine the association between Medicaid expansion and the receipt of inpatient elective bariatric surgery among Medicaid-covered and uninsured individuals aged 26 to 64 years.

**Design, Setting, and Participants:**

This cohort study used difference-in-differences analysis of all-payer data (2010-2017) of 637 557 elective bariatric surgeries for patients aged 26 to 74 years from 11 Medicaid expansion states and 6 nonexpansion states. Nonexpansion states and individuals aged 65 to 74 years were control cohorts. Data analysis was performed from July 6, 2020, to July 23, 2021.

**Exposure:**

Living in a Medicaid expansion state.

**Main Outcomes and Measures:**

The main outcomes were the (1) number of elective bariatric surgeries, (2) population count, and (3) rate of bariatric surgery (number of surgeries per 10 000 population) among Medicaid-covered and uninsured individuals.

**Results:**

Of the 600 798 elective bariatric surgeries in adults aged 26 to 64 years between 2010 and 2017 from the 17 study states, Medicaid-covered and uninsured individuals accounted for 18.3% of the total surgery volume in expansion states and 14.5% in nonexpansion states. A total of 296 798 patients (78.9%) in expansion states were women vs 177 386 (78.9%) in nonexpansion states. Among individuals aged 26 to 64 years, the median age was 44 (IQR, 37-52) years. Racial and ethnic distribution was non-Hispanic White, 60.2%; non-Hispanic Black, 17.7%; Hispanic, 16.6%; and other, 5.5%. Between 2013 and 2017, the volume of bariatric surgeries for Medicaid-covered and uninsured patients increased annually by 30.3% in expansion states and 16.5% in nonexpansion states. Medicaid expansion was associated with a 36.6% annual increase (95% CI, 8.2% to 72.5%) in surgery volume, a 9.0% annual increase (95% CI, 3.8% to 14.5%) in the population, and a 25.5% change (95% CI, –1.3% to 59.4%) in the rate of bariatric surgery. By race and ethnicity, Medicaid expansion was associated with an increase in the rate of bariatric surgery among non-Hispanic White individuals (31.6%; 95% CI, 6.1% to 63.0%) but no significant change among non-Hispanic Black (5.9%; 95% CI, –19.8% to 39.9%) and Hispanic (28.9%; 95% CI, –24.4% to 119.8%) individuals.

**Conclusions and Relevance:**

This cohort study found that Medicaid expansion was associated with increased rates of bariatric surgery among lower-income non-Hispanic White individuals, but not among Hispanic and non-Hispanic Black individuals.

## Key Points

**Question:**

Was Affordable Care Act Medicaid expansion associated with increased uptake of elective bariatric surgery?

**Findings:**

In this cohort study using a difference-in-differences analysis of 637 557 bariatric surgeries from 2010 to 2017 from 11 states that expanded Medicaid and 6 states that did not, Medicaid expansion was associated with a 31.6% annual increase in the rate of bariatric surgery during 2014-2017 among Medicaid-covered and uninsured non-Hispanic White adults aged 26 to 64 years. No increase was observed among non-Hispanic Black and Hispanic adults.

**Meaning:**

This study suggests that additional policy changes and clinical programs may be necessary to address barriers disproportionately faced by racial and ethnic minority populations to ensure more equitable access to evidence-based treatment of obesity.

## Teaser

This cohort study compares the rate of elective bariatric surgery in states with vs without Medicaid expansion after implementation of the Affordable Care Act.

## Introduction

Beginning in 2014, the Affordable Care Act (ACA) state-level Medicaid expansions have been associated with substantial reductions in the uninsured population.^1^ As of 2018, among individuals with low income, the proportion without health insurance was nearly double in nonexpansion states (32%) compared with the expansion states (17%).^2^ Although there is strong evidence that Medicaid expansion was associated with increases in self-reported indicators of health care access, direct evidence of health care utilization has been limited to specific geographic areas and a short follow-up period, particularly for high-cost elective surgeries.^1,3,4,5^

As an elective procedure with most recipients younger than 65 years, bariatric surgery is a marker of health care access to both primary and specialist clinicians.^6^ The rapid increase and high rate of obesity, particularly among populations with low incomes and who are underserved, has exacerbated disease burden, low quality of life, premature death, and health care costs.^7,8,9^ Bariatric surgery is a safe and effective treatment for eligible individuals with obesity, resulting in substantial and lasting weight loss, remission of comorbidities, and reduction in mortality risk.^6,10,11,12,13,14^ Nevertheless, few eligible patients (<1%) undergo bariatric surgery and, among the recipients, those with low incomes and from specific racial and ethnic groups have been shown to be underrepresented.^15^ Although uninsured patients form 16% of the people who are eligible for bariatric surgery, they account for only 0.3% of surgery recipients.^15^ Besides a lack of health insurance, underuse of bariatric surgery has been associated with referral hesitation among primary care clinicians, patient preference for nonsurgical options, rigid treatment protocols, and stringent insurance policy requirements.^6,16,17,18,19^ The ACA Medicaid expansion offers a natural experimental opportunity to evaluate the independent role of insurance in the use of bariatric surgery.^20^ Because many states have not expanded Medicaid following the ACA (24 states in 2014 and 12 states as of August 2021), a large coverage gap has existed between expansion and nonexpansion states for individuals with low income.^21^ To date, evidence of changes in bariatric surgery following Medicaid expansion is limited to 1 study that showed that, during 2014-2015, surgical volume increased in 2 expansion states (Kentucky and Maryland) 10.7% more than in 2 comparison states (Florida and North Carolina).^22^

To develop nationally generalizable evidence of changes in the volume and rate of bariatric procedure use among adults aged 26 to 64 years with a longer period of observation, we used comprehensive inpatient discharge records from 2010 to 2017, including in 17 states that account for nearly 63% of the national population. We incorporated longitudinal changes in the population of Medicaid-covered and uninsured adults to measure the rate of bariatric procedures.

## Methods

### Data and Study Population

We obtained inpatient discharge data from 11 Medicaid expansion states (Arizona, Arkansas, California, Colorado, Illinois, Iowa, Kentucky, New Jersey, New York, Oregon, and Pennsylvania) and 6 states that did not expand Medicaid before December 31, 2017 (Florida, Georgia, North Carolina, Texas, Virginia, and Wisconsin) (eFigure 1 in the Supplement).^23,24^ We obtained 2010-2017 data for all states, with the exception of 3 states for which some years of data were unavailable (2010 and 2011 for Wisconsin and 2017 for Arkansas and New York). States were selected based on population size, regional location, and data availability. These states accounted for 63% of the national population (eTable 1 in the Supplement).^25^ Each state database contains all hospitalization discharge records covering insured and uninsured patients for all short-term acute care hospitals, except for federally owned hospitals (eMethods in the Supplement). We obtained state-level annual data on census population by insurance type from the American Community Surveys.^25,26,27^ This study followed the Strengthening the Reporting of Observational Studies in Epidemiology (STROBE) reporting guideline for cohort studies. The use of these study data was approved by the institutional review board at the Wake Forest School of Medicine. Because this study used deidentified secondary data, informed patient consent was waived by the institutional review board at the Wake Forest School of Medicine.

### Measures

Our main outcomes were bariatric surgery volume, population count, and rate of bariatric surgery among Medicaid-covered and uninsured adults aged 26 to 64 years from 2010 to 2017. We used longitudinal population count adults by insurance type to measure the differential shifts in the uninsured to Medicaid and private coverage in expansion and nonexpansion states.^28^ The rate of bariatric surgery was defined as the number of surgeries per 10 000 population by insurance type. We excluded individuals aged 18 to 25 years because they were the target beneficiaries of the ACA dependent-care expansion introduced in 2010.^1^ We identified bariatric surgeries using *International Classification of Diseases, Ninth Revision, Clinical Modification* (*ICD-9-CM*) and procedure codes for data from January 1, 2010, to September 30, 2015, and the *International Statistical Classification of Diseases, Tenth Revision* and *ICD-10, Clinical Modification* (*ICD-10-CM*) and *ICD-10-Procedure Coding System* (*ICD-10-PCS*) for data from October 1, 2015, to December 31, 2017 (eMethods and eTable 2 in the Supplement). We examined a comprehensive set of bariatric surgeries that included Roux-en-Y gastric bypass, vertical sleeve gastrectomy, and gastric banding, and excluded procedure codes that indicated possible revision surgeries or treatment of complications from an earlier index bariatric surgery.^29,30,31^ We excluded cases with evidence of possible surgical indications other than obesity, such as abdominal procedures for malignant neoplasms and abdominal ulcers.^29^ To identify elective surgeries, we excluded patients admitted through the emergency department. Because our data are by individual state, we excluded out-of-state patients because they are not the target beneficiaries of state-specific Medicaid expansion. We examined outcomes by age, sex, and race and ethnicity (non-Hispanic White [hereafter White], non-Hispanic Black [hereafter Black], Hispanic, and other). We used the combined categorization of race and ethnicity into these 4 groups. This categorization was developed by the Agency for Healthcare Research and Quality, and the combined categorization field was included in the raw data we obtained (eMethods, eTable 2, and eTable 3 in the Supplement report race and ethnicity data and study cohort exclusions).^32^

### Statistical Analysis

We examined the 3 main outcome measures by primary insurance coverage (Medicaid, private, and uninsured). Our focus was the change in the outcome measures associated with ACA Medicaid expansion. We used a 3-way difference-in-differences study design comparing the longitudinal change in each outcome measure in the 11 study states that expanded Medicaid with that in the 6 nonexpansion states and among same-state residents aged 65 to 74 years (bariatric surgery was rare among those aged ≥75 years). Because those older than 65 years were not the target of Medicaid expansion, they were included as comparison cohort controls for within-state secular changes unrelated to expansion (eg, temporal shifts in the type of bariatric surgery used).^1,33,34^ To assess the validity of the comparison, we examined summary trends in the outcomes over time by insurance coverage, age, and expansion status. For our main analysis, we estimated log-linear regression models with a difference-in-differences specification to estimate the percentage change in postexpansion surgery volume associated with expansion (eMethods in the Supplement).^35,36^ Medicaid expansion occurred on January 1, 2014, in 10 of the expansion states and on January 1, 2015, in Pennsylvania. For comparability across states, we defined the year preceding the expansion year as the base year (2014 in Pennsylvania and 2013 in other states) and identified the individual years before and after the base year as relative year dichotomous indicators. We used an event study specification to accommodate the staggered expansion and estimate heterogeneity in changes by relative year.^37,38^ As covariates in the regression model, we included indicators of relative year, state expansion status, and ages 26 to 64 years, along with all 2- and 3-way interactions. In addition, we included state-level fixed effects and obtained SEs clustered at the state level. The estimated change in surgery volume associated with Medicaid expansion is obtained as 100 × (exp[coefficient] − 1) and denotes the percentage change in surgery volume in the expansion states among those aged 26 to 64 years relative to those aged 65 to 75 years within each state and those aged 26 to 64 in nonexpansion states. The coefficients of the 3-way interaction terms indicate the percentage change associated with expansion in outcome measure each year, relative to that in the base year.^37^ To obtain estimates of the overall change in the 4-year postexpansion period (2014-2017), we used similar models with an indicator of the combined postexpansion period in place of the individual years. We estimated the models by insurance coverage and for subgroups by race, ethnicity, age, and sex.^39^ Data processing and all statistical analyses were conducted using Stata, version 16.1 (StataCorp LLC) from July 6, 2020, to July 23, 2021. Tests of model estimates were 2-sided, with significance assessed at *P* ≤ .05.

As a test of the validity of the difference-in-differences design, the above-described models provide estimates to test whether the change before Medicaid expansion (2010 to 2013) was similar among the expansion and nonexpansion states (parallel trends test). To assess the robustness of the estimates, we performed a variety of sensitivity analyses. First, as an alternative to the log-linear models, we estimated the linear and Poisson model analogs. Second, because the popularity of different bariatric procedure types and the availability of new procedure codes were changing during our study period, differential coding for bariatric surgery in the discharge record between areas could affect surgery counts. We used an alternative identification of surgery with only the top 2 procedure codes by frequency (1 each for Roux-en-Y gastric bypass and vertical sleeve gastrectomy) used in all the study years.

## Results

In this cohort study of US patients undergoing bariatric surgery before and after the ACA Medicaid expansion, we examined 637 557 bariatric surgeries performed for patients aged 26 to 74 years during 2010-2017 in the 17 study states (Table 1). Among patients aged 26 to 64 years, 79 255 (21.1%) were men and 296 798 (78.9%) women in the expansion states and 47 359 men (21.1%) and 177 386 women (78.9%). Among patients in all study states, the median age was 44 (IQR, 37-52) years, and the racial and ethnic distribution was Black, 17.7%; Hispanic, 16.6%; White, 60.2%; and other, 5.5%. Among the surgeries for patients aged 26 to 64 years, the combined share of Medicaid-covered and uninsured patients was 18.3% in the expansion states and 14.5% in the nonexpansion states. Compared with surgery recipients with private insurance, higher proportions of Medicaid-covered recipients were younger, women, and Black or Hispanic than their privately covered counterparts. Compared with Medicaid recipients, a smaller proportion of uninsured recipients were Black or Hispanic.

**Table 1.** Demographic Characteristics of 637 557 Patients Undergoing Bariatric Surgery, 2010-2017^a^

Characteristic	No. (%)^b^							
	Medicaid expansion states (n = 11)^c^				Medicaid nonexpansion states (n = 6)^d^			
	All	Payer			All	Payer		
		Medicaid	Uninsured	Private		Medicaid	Uninsured	Private
Bariatric surgeries^e^	395 289 (100)	65 338 (16.5)	7234 (1.8)	261 797 (66.2)	242 268 (100)	14 666 (6.1)	18 001 (7.4)	157 238 (64.9)
Age, y								
26-64	376 053 (100)	65 338 (17.4)	7234 (1.9)	261 797 (69.6)	224 745 (100)	14 666 (6.5)	18 001 (8.0)	157 238 (70.0)
65-74^f^	19 236 (100)	NA	NA	NA	17 523 (100)	NA	NA	NA
**Age 26-64 y^g^**								
Age								
26-34	73 879 (19.6)	21 559 (33.0)	1417 (19.6)	46 786 (17.9)	39 592 (17.6)	4508 (30.7)	3347 (18.6)	28 239 (18.0)
35-44	119 313 (31.7)	23 238 (35.6)	2260 (31.2)	84 874 (32.4)	73 034 (32.5)	5754 (39.2)	5751 (31.9)	53 342 (33.9)
45-54	113 314 (30.1)	14 798 (22.6)	2272 (31.4)	82 306 (31.4)	68 268 (30.4)	3081 (21.0)	5531 (30.7)	47 987 (30.5)
55-64	69 547 (18.5)	5743 (8.8)	1285 (17.8)	47 831 (18.3)	43 851 (19.5)	1323 (9.0)	3372 (18.7)	27 670 (17.6)
Sex								
Male	79 255 (21.1)	8810 (13.5)	1673 (23.1)	58 931 (22.5)	47 359 (21.1)	1572 (10.7)	4823 (26.8)	33 479 (21.3)
Female	296 798 (78.9)	56 528 (86.5)	5561 (76.9)	202 866 (77.5)	177 386 (78.9)	13 094 (89.3)	13 178 (73.2)	123 759 (78.7)
Race and ethnicity								
Hispanic	66 350 (17.6)	18 005 (27.6)	1335 (18.5)	41 055 (15.7)	33 413 (14.9)	3604 (24.6)	3977 (22.1)	21 736 (13.8)
Non-Hispanic Black	55 438 (14.7)	12 846 (19.7)	366 (5.1)	35 092 (13.4)	51 169 (22.8)	5005 (34.1)	1143 (6.3)	35 365 (22.5)
Non-Hispanic White	232 715 (61.9)	29 553 (45.2)	4853 (67.1)	171 758 (65.6)	129 104 (57.4)	5565 (37.9)	11 828 (65.7)	93 087 (59.2)
Other^h^	17 870 (4.8)	4256 (6.5)	623 (8.6)	11 343 (4.3)	8594 (3.8)	346 (2.4)	931 (5.2)	5605 (3.6)
Missing/unknown	3680 (1.0)	678 (1.0)	57 (0.8)	2549 (1.0)	2465 (1.1)	146 (1.0)	122 (0.7)	1445 (0.9)

Abbreviation: NA, not applicable.

^a^ Data on 2010-2011 for Wisconsin and 2017 for Arkansas and New York were missing.

^b^ The column All includes individuals with any payer/insurance type. In addition to Medicaid, uninsured, and private, the main other payer is Medicare (among individuals <65 years). Therefore, the sums of counts by payer do not add to the All count.

^c^ Arizona, Arkansas, California, Colorado, Illinois, Iowa, Kentucky, New Jersey, New York, Oregon, and Pennsylvania.

^d^ Florida, Georgia, North Carolina, Texas, Virginia, and Wisconsin.

^e^ The percentages are row-wise share by payer group, separately for expansion and nonexpansion states.

^f^ Patients were presumed to have Medicare coverage.

^g^ Percentages are column-wise share (eg, all race and ethnicity percentage figures for each column group total 100).

^h^ Asian, American Indian, Native Hawaiian and other Pacific Islander, and multiracial groups, categorized in accordance with the Agency for Healthcare Research and Quality.^32^

### Observed Changes

By the third year after expansion, the volume of bariatric surgeries for the Medicaid-covered and uninsured cohorts increased by 90.9% in the expansion states and 49.5% in the nonexpansion states (Figure; eFigure 2 and eTable 4a in the Supplement). Although the increase was similar among the Medicaid-covered (50.0%) and uninsured (49.1%) individuals in nonexpansion states, in the expansion states, Medicaid-covered volume increased 103.5%, and uninsured volume increased 9.9%. Among those aged 65 to 74 years (and presumed to be covered by Medicare), the surgery volume increased 16.7% in the expansion states and 26.8% in the nonexpansion states.

**Figure.**
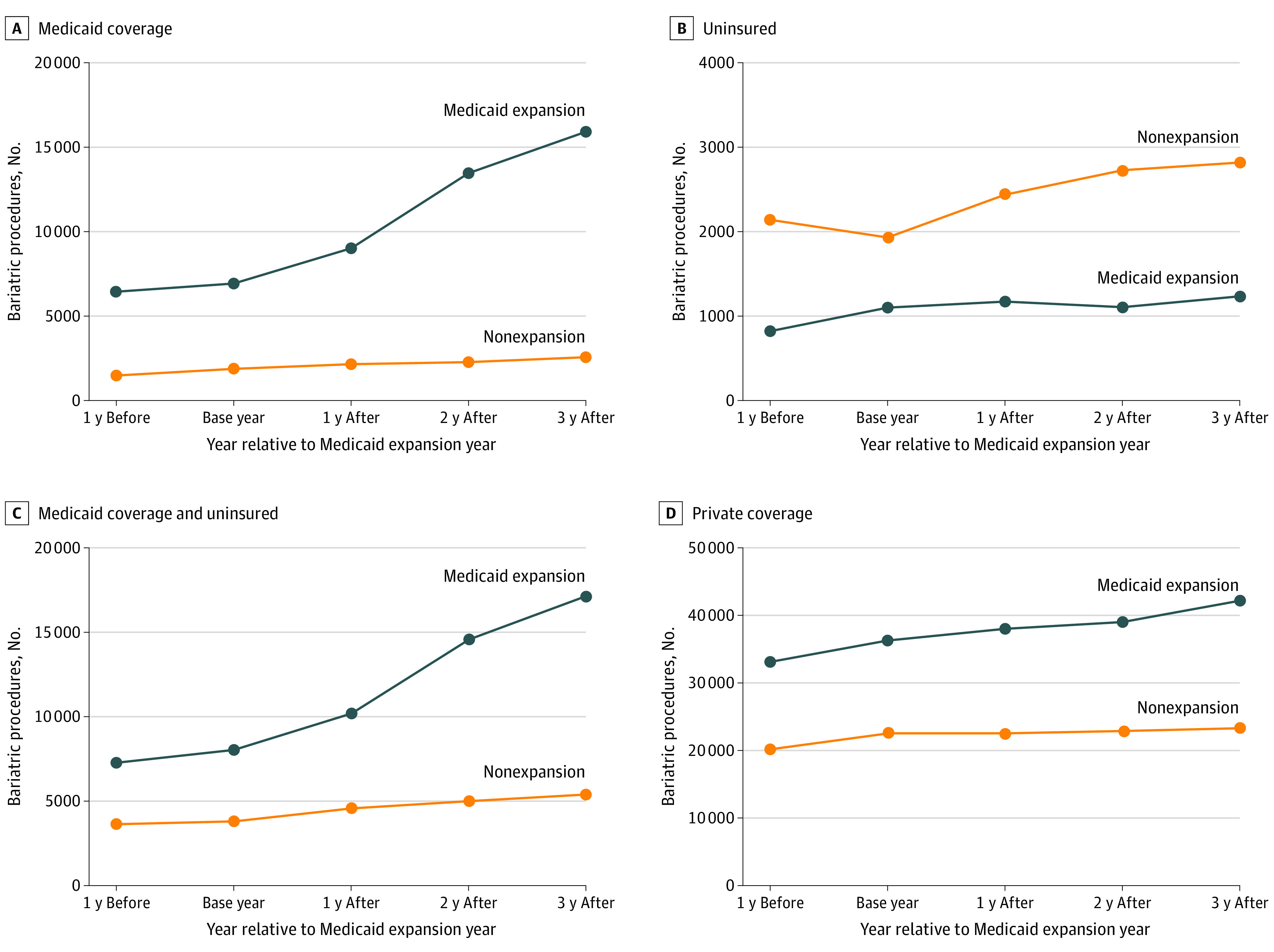
Annual Change in the Aggregate Number of Bariatric Surgeries by Insurance Coverage, 2010-2017 Observed count of bariatric surgeries by insurance coverage from each state were aggregated for Medicaid expansion and nonexpansion states separately for individuals with Medicaid (A), those uninsured (B), Medicaid coverage combined with uninsured (C), and those with private insurance (D). Data for all years (2010-2017) were not available for all 17 study states. Specifically, 2010-2011 data for Wisconsin and 2017 data for Arkansas and New York were missing. As a result, 3 years before and 3 years after the base year for all states except Wisconsin are shown. eTable 4 in the Supplement supplies the counts by relative year and calendar year.

### Change Associated With Medicaid Expansion by Insurance Coverage

Medicaid expansion was associated with an increase in surgery volume for Medicaid-covered and uninsured patients by 42.8% (95% CI, 10.6% to 84.3%) in the second year after expansion and 43.8% (95% CI, 9.3% to 89.3%) in the third year (Table 2; eTable 4b in the Supplement). Over the 4-year period (2014-2017), the annual change was 36.6% (95% CI, 8.2% to 72.5%). Although the volume for Medicaid-covered individuals increased 45.8% (95% CI, 6.2% to 100.1%), that for uninsured patients was not significant (−10.6%; 95% CI, –42.7% to 39.5%). The surgery volume change among privately insured patients was also not significant (8.0%; 95% CI, –13.6% to 35.0%).

**Table 2.** Percentage Change in Bariatric Surgeries Associated With Medicaid Expansion: Overall and by Patient Insurance Coverage^a^

Insurance payer	Aggregate No. bariatric procedures, base year (age 26-64 y)		Model 1: change in bariatric surgery volume by postreform year, % (95% CI)^b^^,^^c^							Model 2: annual postreform change, % (95% CI)^b,c^
	ES	NES	3rd Year before base year	2nd Year before base year	1 y Before base year	Year 1 after expansion	Year 2 after expansion	Year 3 after expansion	Year 4 after expansion	Years 1-4 after expansion
Medicaid and uninsured	7638	3507	–37.8 (–67.4 to 18.7)	–14.2 (–63.7 to 102.9)	–5.6 (–53.3 to 90.9)	13.4 (–7.3 to 38.8)	42.8 (10.6 to 84.3)^d^	43.8 (9.3 to 89.3)^d^	21.9 (–29.1 to 109.6)	36.6 (8.2 to 72.5)^^d^^
Medicaid	6609	1717	–29.8 (–69.2 to 59.6)	2.8 (–62.2 to 179.3)	16.1 (–41.7 to 131.2)	29.7 (–3.1 to 73.5)	71.2 (28.9 to 127.5)^d^	60.7 (11.4 to 132.0)^d^	29.6 (–28.1 to 133.5)	45.8 (6.2 to 100.1)^^d^^
Uninsured	1029	1790	–33.1 (–59.3 to 9.9)	–43.6 (–72.1 to 13.7)	–28.1 (–62.0 to 36.3)	1.3 (–35.6 to 59.4)	–17.0 (–46.8 to 29.4)	–15.9 (–45.4 to 29.7)	–9.1 (–52.8 to 75.3)	–10.6 (–42.7 to 39.5)
Private coverage	33 319	21 138	–18.1 (–38.7 to 9.5)	–3.1 (–29.8 to 33.8)	–11.1 (–32.8 to 17.6)	6.6 (–10.1 to 26.5)	10.9 (–11.7 to 39.2)	12.0 (–13.2 to 44.5)	0.4 (–30.6 to 45.2)	8.0 (–13.6 to 35.0)
All payers	46 295	28 827	–19.3 (–38.8 to 6.4)	–5.0 (–30.4 to 29.6)	–14.7 (–39.6 to 20.3)	8.2 (–7.2 to 26.3)	19.0 (–2.1 to 44.6)	20.2 (–4.2 to 50.8)	2.9 (–30.8 to 53.0)	17.0 (–4.0 to 42.5)

Abbreviations: ES, expansion states; NES, nonexpansion states.

^a^ The estimates in each row are from a separate regression using observations for the respective payer groups. Model 1 estimates for all years are included, but for model 2, only the estimate for the composite postreform period (years 1-4 after expansion) is reported. The full regression model 1 estimates are in eTable 4b in the Supplement; because model 2 estimates are similar for the common covariates, they are not reported. Each regression was based on 264 observations, consisting of 17 states × (up to) 8 years × 2 age groups (26-64 and 65-74 years). As noted in Table 1, 8 years of data were available for all states, except Wisconsin (6 years) and Arkansas and New York (7 years). Estimates reported here are from the log-linear regression model specification. Corresponding estimates from other model specifications are in eTable 7 in the Supplement.

^b^ The estimated change in surgery volume associated with Medicaid expansion was obtained as 100 × (exp[coefficient] – 1) and denotes the percentage change in surgery volume in the expansion states among individuals aged 26 to 64 years compared with those aged 65 to 75 years within each state and those aged 26 to 64 years in nonexpansion states. The estimates of percentage change in bariatric surgery volume use the base year as the reference year. Base year is the year preceding the expansion year, which is 2013 for all states except Pennsylvania, for which 2014 was the base year. The percentage change estimates reflect the change associated with Medicaid expansion. See the eMethods in the Supplement for the model specification details.

^c^ The 95% CIs were obtained based on SEs clustered at the state level.

^d^ Significant at *P* < .05 level.

### Change in Medicaid-Covered and Uninsured Population

By the third year after expansion, in the expansion states, the uninsured population aged 26 to 64 years decreased by 5.5 million, the Medicaid-covered population increased by 3.8 million, and the private coverage population increased by 2.6 million (eTable 5a and eFigure 3 in the Supplement). The combined Medicaid-covered and uninsured population decreased by 9.3% (1.7 million) in expansion states and 16.6% (2.0 million) in nonexpansion states. The expansion was associated with a 9.0% (95% CI, 3.8% to 14.5%) annual increase in the population count of the Medicaid-covered and uninsured individuals (Table 3 and eTable 5b in the Supplement).

**Table 3.** Change (%) in Census Population by Payer and in Rate of Bariatric Surgery Associated With Medicaid Expansion^a^

Insurance payer	Aggregate census population aged 26-64 y (thousands), base year		Change in census population by reform year, % (95% CI)^c^								Baseline rate of bariatric surgery (No./10 000 population)		Change in rate of bariatric surgery by postreform year, % (95% CI)^c^							
			Model 1: change (%) in census population by postreform year							Model 2: annual postreform change (%)			Model 1: change (%) in rate of bariatric surgery by postreform year							Model 2: annual postreform change (%)
	ES	NES	3rd Year before base year	2nd Year before baseline	1 y Before baseline	Year 1 after expansion	Year 2 after expansion	Year 3 after expansion	Year 4 after expansion	Years 1-4 after expansion	ES	NES	3rd Year before base year	2nd Year before baseline	1 y Before baseline	Year 1 after expansion	Year 2 after expansion	Year 3 after expansion	Year 4 after expansion	Years 1-4 after expansion
Medicaid and uninsured	18 227	13 088	–1.7 (–9.8 to 7.1)	–2.0 (–9.2 to 5.8)	–0.2 (–3.2 to 3.0)	4.8 (2.8 to 6.8)^b^	10.2 (5.3 to 15.3)^b^	10.9 (4.9 to 17.3)^b^	11.8 (–2.6 to 28.3)	9.0 (3.8 to 14.5)^b^	43.2	16.2	–35.2 (–64.2 to 17.5)	–13.3 (–60.9 to 92.6)	–7.9 (–51.2 to 73.9)	8.0 (–10.8 to 30.7)	29.3 (0.7 to 66.0)^b^	30.3 (–0.8 to 71.1)	11.1 (–34.2 to 87.7)	25.5 (–1.3 to 59.4)
Medicaid	6612	2989	4.8 (–8.4 to 19.8)	4.7 (–6.0 to 16.8)	–2.3 (–6.5 to 2.0)	22.7 (13.4 to 32.8)^b^	41.1 (23.1 to 61.7)^b^	44.6 (26.7 to 64.9)^b^	44.7 (14.7 to 82.5)^b^	37.9 (21.2 to 57.0)^b^	92.2	37.1	–31.9 (–66.7 to 39.5)	–2.5 (–60.2 to 138.7)	16.3 (–37.0 to 115.0)	5.4 (–18.3 to 36.0)	20.9 (–3.7 to 51.8)	11.6 (–16.3 to 48.8)	–10.5 (–41.4 to 36.7)	5.7 (–15.1 to 31.6)
Uninsured	11 614	10 100	–2.4 (–15.1 to 12.2)	–3.7 (–15.9 to 10.3)	1.6 (–2.4 to 5.8)	–11.4 (–16.0 to –6.4)^b^	–22.0 (–32.1 to –10.5)^b^	–26.7 (–37.5 to –14.0)^b^	–22.9 (–40.0 to –0.8)^b^	–20.3 (–31.0 to –8.0)^b^	12.6	8.1	–30.6 (–54.3 to 5.3)	–38.0 (–66.5 to 14.7)	–28.5 (–59.6 to 26.7)	7.4 (–25.5 to 54.8)	2.3 (–31.1 to 51.8)	8.6 (–26.2 to 59.8)	7.9 (–39.4 to 92.1)	5.8 (–27.0 to 54.5)
Private coverage	41 950	26 596	1.8 (–2.3 to 6.2)	0.8 (–2.8 to 4.5)	–0.0 (–1.6 to 1.5)	–2.4 (–4.3 to –0.5)^b^	–5.3 (–8.6 to –1.9)^b^	–5.4 (–9.5 to –1.0)^b^	–3.9 (–9.3 to 1.9)	–4.1 (–7.4 to –0.6)^b^	78.3	42.9	–18.7 (–37.6 to 6.0)	–3.7 (–29.1 to 30.8)	–10.7 (–32.1 to 17.5)	9.1 (–7.0 to 28.0)	16.6 (–5.6 to 44.0)	18.1 (–7.0 to 50.0)	8.8 (–17.9 to 44.2)	13.2 (–7.2 to 38.1)
All payers	61 271	40 599	0.7 (–2.8 to 4.2)	–0.3 (–3.0 to 2.5)	–0.2 (–1.5 to 1.2)	–0.1 (–0.9 to 0.7)	–0.7 (–1.6 to 0.2)	–0.7 (–2.4 to 1.0)	0.6 (–4.4 to 5.9)	–0.3 (–1.8 to 1.3)	77.2	39.7	–19.0 (–36.6 to 3.5)	–4.7 (–28.6 to 27.2)	–14.1 (–38.6 to 20.0)	8.2 (–6.8 to 25.7)	19.3 (–1.0 to 43.8)	20.8 (–3.0 to 50.4)	7.8 (–20.3 to 45.9)	16.9 (–3.2 to 41.2)

Abbreviations: ES, expansion states; NES, nonexpansion states.

^a^ The estimates in each row are from a separate regression using observations for the respective payer groups. Model 1 estimates for all years are included, but for model 2, only the estimate for the composite postreform period (years 1-4 after expansion) is reported. The full regression model 1 estimates are in eTable 5b and eTable 6b in the Supplement; because model 2 estimates are similar for the common covariates, they are not reported. Each regression was based on 264 observations, consisting of 17 states × (up to) 8 years × 2 age groups (26-64 and 65-74 years). As noted in Table 1, 8 years of data were available for all states, except Wisconsin (7 years) and Arkansas and New York (7 years). Estimates reported here from the log-linear regression model specification. The estimated change in surgery volume associated with Medicaid expansion was obtained as 100 × (exp[coefficient] − 1) and denotes the percentage change in surgery volume in the expansion states among individuals aged 26 to 64 years compared with those aged 65 to 75 years within each state and those aged 26 to 64 years in nonexpansion states. The estimates of percentage change in each outcome measure use the base year as the reference year. Base year is the year preceding the expansion year, which is 2013 for all states except Pennsylvania, for which 2014 was the base year. The percentage change estimates reflect the change associated with Medicaid expansion. See the eMethods in the Supplement for the model specification details. The 95% CIs were obtained based on SEs clustered at the state level.

^b^ Significant at *P* < .05 level.

### Change in Rate of Bariatric Surgery

In the base year, in the expansion states, the rate of bariatric surgery was 10.0 surgeries per 10 000 population among Medicaid-covered individuals and 0.9 surgeries per 10 000 population among uninsured individuals; in the nonexpansion states, the corresponding rates were 5.6 surgeries per 10 000 population and 1.8 surgeries per 10 000 population (eTable 6a and eFigure 4 in the Supplement). In the expansion states, the surgery rate for Medicaid-covered and uninsured combined increased from 4.2 per 10 000 population (base year) to 8.8 per 10 000 population 3 years after expansion, amounting to a 110.4% increase; the corresponding change in the nonexpansion states was an increase of 79.2%. Medicaid expansion was associated with a 29.3% increase (95% CI, 0.7% to 66.0%) in the rate of bariatric surgery in the second year after expansion (Table 3; and eTable 6b in the Supplement). The mean annual change during 2014-2017 was not statistically significant (25.5%; 95% CI, –1.3% to 59.4%).

### Change Among Subpopulations

Stratified analyses of Medicaid-covered and uninsured individuals by race and ethnicity indicate that Medicaid expansion was associated with an annual increase of 44.7% (95% CI, 13.5% to 84.4%) among White individuals (Table 4). The corresponding annual change among Black (15.6%; 95% CI, –10.0% to 48.3%) and Hispanic (26.8%; 95% CI, –20.5% to 102.1%) individuals was not statistically significant. The expansion was associated with an increase in Medicaid-covered and uninsured populations among White (10.1%; 95% CI, 3.9% to 16.6%) and Black (12.1%; 95% CI, 5.6% to 18.9%) individuals, but not among Hispanic (6.7%; 95% CI, –1.2% to 15.2%) individuals. Although the rate of bariatric surgery increased among White individuals (31.6%; 95% CI, 6.1% to 63.0%), the change among Black (5.9%; 95% CI, –19.8% to 39.9%) and Hispanic (28.9%; 95% CI, –24.4% to 119.8%) individuals was not significant. Estimation by age groups indicated that the surgery volume increased among those aged 26 to 44 and 45 to 64 years, but the surgery rate increased only among those aged 45 to 64 years (eTable 7a in the Supplement). In addition, estimation by sex indicated increased surgery volume among women (eTable 7b in the Supplement).

**Table 4.** Change (%) by Race/Ethnicity in Bariatric Surgery Volume and Rate Associated With Medicaid Expansion Among Medicaid Covered + Uninsured^a^

Race and ethnicity	Baseline surgery volume/census population (thousands)/surgery rate (No./10 000 population)		Model 1: change in census population by postreform year, % (95% CI)							Model 2: annual postreform change, % (95% CI)
	ES	NES	3rd Year before base year	2nd Year before baseline	1 y Before baseline	Year 1 after expansion	Year 2 after expansion	Year 3 after expansion	Year 4 after expansion	Years 1-4 after expansion
**Volume of bariatric surgery**										
Non-Hispanic White	3200	1924	–29.5 (–60.3 to 25.3)	0.9 (–53.1 to 117.1)	4.9 (–46.4 to 105.4)	22.0 (–7.7 to 61.2)	63.3 (23.5 to 115.8)^b^	64.6 (19.7 to 126.4)^b^	36.8 (–4.1 to 95.2)	44.7 (13.5 to 84.4)^d^
Non-Hispanic Black	1415	693	–2.3 (–62.3 to 152.9)	32.9 (–50.4 to 255.7)	14.3 (–52.5 to 174.9)	16.4 (–19.7 to 68.8)	60.8 (15.3 to 124.3)^b^	23.0 (–1.0 to 52.7)	–33.2 (–67.8 to 38.5)	15.6 (–10.0 to 48.3)
Hispanic	2366	745	–47.2 (–79.2 to 33.9)	–43.3 (–81.1 to 70.2)	–48.8 (–72.4 to –5.0)	2.1 (–35.3 to 61.2)	13.5 (–25.7 to 73.5)	24.9 (–18.3 to 91.0)	81.6 (–15.8 to 291.8)	26.8 (–20.5 to 102.1)
**Census population of Medicaid covered + uninsured**										
Non-Hispanic White	7349.8	5243.9	1.7 (–4.8 to 8.7)	1.6 (–3.4 to 6.9)	–0.6 (–4.6 to 3.5)	6.4 (3.5 to 9.4)^b^	11.2 (5.0 to 17.7)^b^	10.5 (3.8 to 17.7)^b^	10.2 (–1.9 to 23.9)	10.1 (3.9 to 16.6)^b^
Non-Hispanic Black	2329.5	2679.5	–7.5 (–12.9 to –1.7)	–4.5 (–9.3 to 0.6)	–3.9 (–10.1 to 2.8)	3.4 (–1.0 to 8.0)	11.4 (3.6 to 19.7)^b^	16.6 (6.8 to 27.2)^b^	13.8 (6.2 to 21.9)	12.1 (5.6 to 18.9^b^
Hispanic	6612.0	4514.3	1.6 (–7.3 to 11.3)	–1.2 (–9.4 to 7.8)	1.4 (–2.8 to 5.9)	2.4 (–3.0 to 8.0)	9.2 (1.7 to 17.3)^b^	7.8 (–0.3 to 16.6)	5.8 (–5.9 to 19.0)	6.7 (–1.2 to 15.2)
**Rate of bariatric surgery**										
Non-Hispanic White	44.8	20.5	–29.8 (–59.0 to 20.0)	–2.1 (–52.5 to 101.8)	2.8 (–44.8 to 91.5)	14.2 (–12.0 to 48.2)	46.2 (15.5 to 85.2)^b^	49.3 (10.3 to 102.2)^b^	25.3 (–12.1 to 78.6)	31.6 (6.1 to 63.0)^d^
Non-Hispanic Black	50.9	18.5	6.9 (–57.0 to 165.8)	36.7 (–44.7 to 238.1)	18.3 (–46.6 to 162.1)	11.4 (–22.4 to 59.9)	49.9 (2.6 to 118.9)^b^	7.2 (–16.4 to 37.5)	–36.4 (–67.4 to 23.9)	5.9 (–19.8 to 39.9)
Hispanic	37.2	7.9	–43.9 (–74.8 to 24.7)	–35.4 (–78.7 to 95.7)	–45.3 (–70.3 to 0.8)	3.4 (–39.0 to 75.3)	4.3 (–37.1 to 73.1)	31.2 (–20.1 to 115.5)	101.8 (–14.9 to 378.5)	28.9 (–24.4 to 119.8)

Abbreviations: ES, expansion states; NES, nonexpansion states.

^a^ The estimates in each row are from a separate regression using observations for the respective payer groups. Model 1 estimates for all years are included, but for model 2, only the estimate for the composite postreform period (years 1-4 after expansion) is reported. The estimates in each row are from a separate regression using observations for the respective race and ethnicity group. Each regression was based on 264 observations, consisting of 17 states × (up to) 8 years × 2 age groups (26-64 and 65-74 years). As noted in Table 1, we had 8 years of data for all states, except Wisconsin (6 years) and Arkansas and New York (7 years). Estimates reported here from the log-linear regression model specification. The estimated change in each of the 3 outcome measures associated with Medicaid expansion was obtained as 100 × (exp[coefficient] – 1) and denotes the percentage change in surgery volume in the expansion states among individuals aged 26 to 64 years compared with those aged 65 to 75 years within each state and those aged 26 to 64 years in nonexpansion states. The estimates of percentage change in each outcome measure used the base year as the reference year. Base year is the year preceding the expansion year, which is 2013 for all states except Pennsylvania (for which 2014 was the base year). The percentage change estimates reflect the change associated with Medicaid expansion. See the eMethods in the Supplement for the model specification details. The 95% CIs were obtained based on SEs clustered at the state level.

^b^ Significant at *P* < .05 level.

### Supplementary and Sensitivity Analysis

As a test of parallel trends, we consistently found that the differences in preexpansion trends in expansion and nonexpansion states were not statistically significant. As a sensitivity analysis, we obtained estimates of change associated with Medicaid expansion using linear and Poisson regression models of the volume in bariatric surgery (eTable 7 and eTable 8 in the Supplement). The results are consistent with those from the log-linear models reported in Table 2. Because types of bariatric surgery have changed over time, their use and coding may vary by location. As an alternative, we measured volume using only the 2 most common types and codes of surgery (eMethods in the Supplement). The longitudinal trend of the 2 surgery types was similar in expansion and nonexpansion states (eTable 9a in the Supplement). The estimates of the change in surgery volume associated with Medicaid expansion were consistent with those using all types of bariatric surgery (eTable 9b in the Supplement).

## Discussion

In this cohort study of US patients undergoing bariatric surgery, the ACA Medicaid expansion was associated with an annual increase of over 40% in the volume of bariatric surgery during 2014-2017 among adults aged 26 to 64 years with Medicaid coverage and no health insurance. Approximately 9% of the volume growth was attributable to a smaller reduction in the Medicaid-covered and uninsured populations in the states that expanded Medicaid compared with nonexpansion states. This difference suggests that an increase in the surgery rate may account for the remaining increase in surgery volume; however, the direct estimate of the change in the surgery rate associated with expansion was statistically significant in only 1 of the postreform years. By race and ethnicity, expansion was associated with increased surgery volume and surgery rate among White individuals; the corresponding change among Black and Hispanic individuals was not statistically significant.

Our findings suggest the importance of examining the relative shifts in the uninsured population in the expansion and nonexpansion states. The target population of those with Medicaid coverage and those with no insurance decreased more in nonexpansion states than in expansion states. This change is likely the result of those with household income between 100% and 138% of the federal poverty level being eligible for Medicaid in expansion states and subsidized private coverage in nonexpansion states, leading to a larger proportion of uninsured gaining private coverage in nonexpansion states.^28^ Among White individuals, expansion was associated with a 44.7% increase in surgery volume, a 10.1% increase in population, and a 31.6% increase in surgery rate. In contrast, among Black individuals, expansion was associated with a 12.1% increase in population, but the changes in surgery volume and surgery rate were not statistically significant.

Our findings are consistent with those of a study of bariatric surgery using data from 2012-2015 from 2 expansion states (Kentucky and Maryland) and 2 nonexpansion states (Florida and North Carolina).^22^ The state-level fixed effects specification in our approach provides estimates that are based only on within-state longitudinal changes in surgery use. In contrast, the estimates from the earlier study were based on variation from longitudinal changes and cross-sectional differences in use patterns, and therefore are susceptible to confounding from differences in baseline surgery levels in expansion and nonexpansion states.^40^

Many factors previously identified may be associated with the absence of significant change in surgery volume and rate among Black and Hispanic individuals.^41,42^ Noninsurance barriers may differentially affect Black and Hispanic patients with Medicaid coverage. Previous studies noted that Black and Hispanic patients eligible for surgery reported a higher rate of willingness to undergo surgery if recommended by their physician relative to White patients; however, Black and Hispanic patients were less likely to be referred to surgery.^43^ It is possible that surgical evaluation of potentially eligible patients and the preapproval process may exclude more Black and Hispanic patients for not meeting presurgical lifestyle change or weight loss requirements.^44^ Disparities in the change in surgery use are likely associated with the role of pervasive systemic and structural racism.^45,46^ At the health system level, and particularly among surgeons, there is a lack of workforce diversity, and clinicians may hold explicit or implicit racial biases that may result in under-referral of Black patients to bariatric surgery.^47,48^ Ongoing racism and discrimination impede patients from seeking and receiving appropriate care.^45^

### Limitations

This study has limitations. First, other sources of unobserved confounding cannot be ruled out, particularly from other policy changes that occurred at the state and national levels.^1,36^ Our estimates are robust to secular changes (eg, practice patterns or surgery options). Second, our estimates have wide CIs due to SEs clustered at the state level and so are indeterminate for tests of sizable estimates. In the case of prereform trends, wide CIs may fail to identify the absence of parallel trends for some outcomes examined. Third, our data contain no indicators for the appropriateness of bariatric surgery. However, insurers generally mandate screening based on lifestyle modification and counseling before surgery approval. Fourth, a change in the coding of discharge records from *ICD-9-CM* to *ICD-10-CM* and *ICD-10-PCS* may contribute to artificial shifts in volume counts. We found that change in the observed counts immediately before and after the switch to *ICD-10-CM or ICD-10-PCS* on October 1, 2015, is small. Our data did not include the increasing share of bariatric surgeries in the ambulatory setting (8.1% of aggregate volume in 2017), particularly for vertical-sleeve gastrectomy procedures.^49^ It is unclear whether rates of bariatric surgeries performed in ambulatory centers differ substantially between expansion and nonexpansion states, particularly among low-income patients.

## Conclusions

In this study, Medicaid expansion was associated with an increase in the volume and rate of bariatric surgery among lower-income White individuals, but not among Hispanic and Black patients. Providing insurance coverage may remove one barrier faced by patients in accessing bariatric surgery. Additional policy changes and clinical programs may be necessary to address barriers disproportionately faced by racial and ethnic minority populations to ensure more equitable access to evidence-based treatment of obesity.

## Notes

Supplement. **eFigure 1.** Study States by Expansion Status **eTable 1.** Share of the Study States in the National Census Population Aged 18-64 Years by Race/Ethnicity, 2012 **eMethods.** Detailed Methods **eTable 2.** Identification of Gastric Surgeries **eTable 3.** Cohorts and Counts of Bariatric Surgeries **eFigure 2.** Longitudinal Volume of Bariatric Surgeries by Insurance Payer and State Medicaid Expansion Status **eTable 4.** Number and Changes in Bariatric Surgeries **eFigure 3.** Longitudinal Volume of Census Population by Insurance Payer and State Medicaid Expansion Status **eTable 5.** Number and Changes in Census Population **eFigure 4.** Longitudinal Rate of Bariatric Surgery by Insurance Payer and State Medicaid Expansion Status **eTable 6.** Rate and Change in Bariatric Surgery **eTable 7.** Change by Age and Sex in Bariatric Surgery Volume and Rate **eTable 8.** Linear and Poisson Models of Change **eTable 9.** Bariatric Surgery by Type
