# Supplementary material for: Examination of Elective Bariatric Surgery Rates Before and After US Affordable Care Act Medicaid Expansion
Source: JAMA Health Forum. 2021 Oct 8;2(10):e213083. doi: 10.1001/jamahealthforum.2021.3083 (PMC8727038; doi:10.1001/jamahealthforum.2021.3083)
Supplement: Supplement. — eFigure 1. Study States by Expansion Status eTable 1. Share of the Study States in the National Census Population Aged 18-64 Years by Race/Ethnicity, 2012 eMethods. Detailed Methods eTable 2. Identification of Gastric Surgeries eTable 3. Cohorts and Counts of Bariatric Surgeries eFigure 2. Longitudinal Volume of Bariatric Surgeries by Insurance Payer and State Medicaid Expansion Status eTable 4. Number and Changes in Bariatric Surgeries eFigure 3. Longitudinal Volume of Census Population by Insurance Payer and State Medicaid Expansion Status eTable 5. Number and Changes in Census Population eFigure 4. Longitudinal Rate of Bariatric Surgery by Insurance Payer and State Medicaid Expansion Status eTable 6. Rate and Change in Bariatric Surgery eTable 7. Change by Age and Sex in Bariatric Surgery Volume and Rate eTable 8. Linear and Poisson Models of Change eTable 9. Bariatric Surgery by Type [file jamahealthforum-e213083-s001.pdf]

Supplementary Online Content

Hanchate AD, Qi D, Paasche-Orlow MK, et al. Examination of elective bariatric surgery rates before and after US Affordable Care Act Medicaid expansion. *JAMA Health Forum*. 2021;2(10):e213083. doi:10.1001/jamahealthforum.2021.3083

eFigure 1. Study States by Expansion Status

eTable 1. Share of the Study States in the National Census Population Aged 18-64 Years by Race/Ethnicity, 2012

eMethods. Detailed Methods

eTable 2. Identification of Gastric Surgeries

eTable 3. Cohorts and Counts of Bariatric Surgeries

eFigure 2. Longitudinal Volume of Bariatric Surgeries by Insurance Payer and State Medicaid Expansion Status

eTable 4. Number and Changes in Bariatric Surgeries

eFigure 3. Longitudinal Volume of Census Population by Insurance Payer and State Medicaid Expansion Status

eTable 5. Number and Changes in Census Population

eFigure 4. Longitudinal Rate of Bariatric Surgery by Insurance Payer and State Medicaid Expansion Status

eTable 6. Rate and Change in Bariatric Surgery

eTable 7. Change by Age and Sex in Bariatric Surgery Volume and Rate

eTable 8. Linear and Poisson Models of Change

eTable 9. Bariatric Surgery by Type

This supplementary material has been provided by the authors to give readers additional information about their work.

eFigure 1. Study states by expansion status

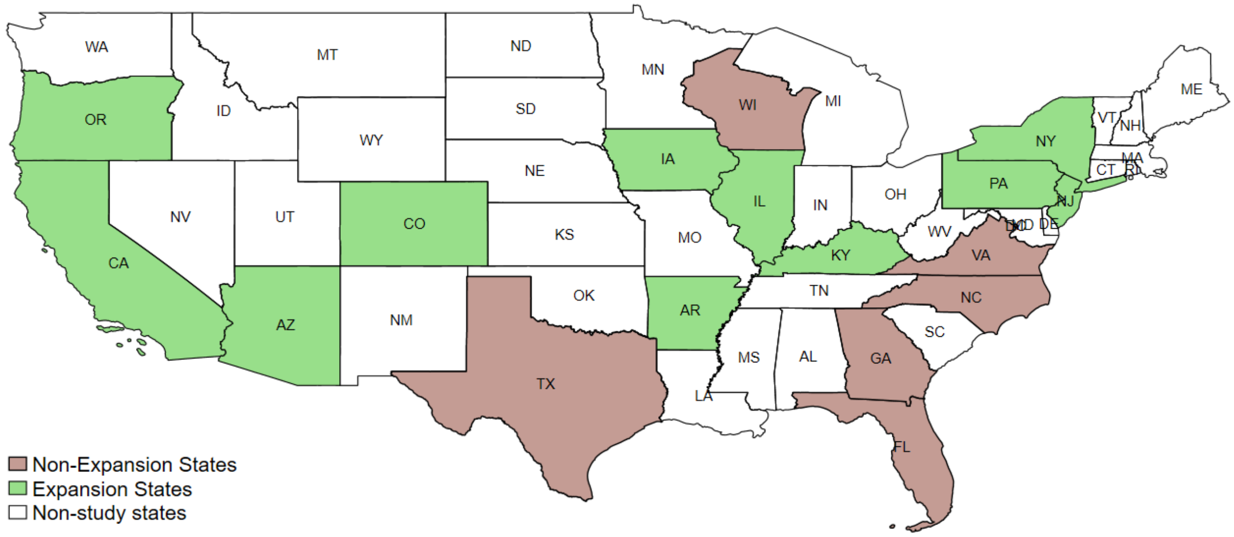

Note:
1) Medicaid expansion status was as of 9/30/2015. Of the 11 expansion states, 10 expanded on 1/1/2014 and Pennsylvania expanded on 1/1/2015.

eTable 1. Share of the study states in the national census population aged 18-64 years by race/ethnicity, 2012

State	All	White, non-Hispanic	Black, non-Hispanic	Hispanic
Study States (N=17)				
AZ	2.01%	1.15%	0.09%	0.59%
AR	0.91%	0.67%	0.14%	0.06%
CA	12.43%	5.03%	0.79%	4.55%
CO	1.70%	1.21%	0.07%	0.33%
FL	5.97%	3.29%	0.98%	1.46%
GA	3.17%	1.75%	0.99%	0.27%
IL	4.09%	2.60%	0.59%	0.64%
IA	0.95%	0.84%	0.03%	0.05%
KY	1.38%	1.19%	0.12%	0.04%
NJ	2.83%	1.61%	0.38%	0.54%
NY	6.38%	3.61%	0.96%	1.19%
NC	3.09%	2.01%	0.68%	0.25%
OR	1.26%	0.99%	0.03%	0.14%
PA	4.02%	3.17%	0.45%	0.25%
TX	8.19%	3.73%	1.01%	2.99%
VA	2.67%	1.71%	0.52%	0.23%
WI	1.80%	1.51%	0.12%	0.10%
All study states (N=17)	62.87%	36.09%	7.95%	13.68%
Non-Study States (N=33)				
AL	1.51%	1.00%	0.41%	0.06%
AK	0.25%	0.17%	0.01%	0.01%
CT	1.15%	0.80%	0.12%	0.16%
DE	0.29%	0.19%	0.06%	0.02%
DC	0.23%	0.09%	0.10%	0.02%
HI	0.56%	0.16%	0.01%	0.06%
ID	0.48%	0.41%	0.00%	0.05%
IN	2.04%	1.67%	0.19%	0.12%
KS	0.90%	0.70%	0.06%	0.09%
LA	1.45%	0.88%	0.46%	0.07%
ME	0.42%	0.40%	0.01%	0.01%
MD	1.92%	1.03%	0.58%	0.17%
MA	2.17%	1.64%	0.15%	0.22%
MI	3.12%	2.40%	0.45%	0.13%
MN	1.70%	1.42%	0.10%	0.08%
MS	0.92%	0.53%	0.35%	0.03%
MO	1.89%	1.52%	0.23%	0.07%
MT	0.32%	0.28%	0.00%	0.01%
NE	0.57%	0.47%	0.03%	0.05%
NV	0.89%	0.48%	0.08%	0.23%

NH	0.43%	0.39%	0.01%	0.01%
NM	0.65%	0.26%	0.01%	0.30%
ND	0.22%	0.20%	0.00%	0.01%
OH	3.62%	2.94%	0.46%	0.11%
OK	1.22%	0.85%	0.10%	0.11%
RI	0.34%	0.26%	0.02%	0.04%
SC	1.48%	0.95%	0.42%	0.08%
SD	0.26%	0.22%	0.00%	0.01%
TN	2.03%	1.53%	0.35%	0.09%
UT	0.86%	0.69%	0.01%	0.11%
VT	0.20%	0.19%	0.00%	0.00%
WA	2.27%	1.64%	0.10%	0.24%
WV	0.58%	0.54%	0.02%	0.01%
WY	0.18%	0.16%	0.00%	0.02%
All non-study states (N=33 & DC)	37.13%	27.04%	4.90%	2.81%
All states (N=50 & DC)	100.00%	63.13%	12.86%	16.49%
Count of population	201,136,985	126,975,739	25,861,075	33,163,609

Notes:
1) Source: U.S. Census Bureau (2020). State Population Totals and Components of Change: 2010-2019. Washington, DC, U.S. Census Bureau.
2) Each cell in the above table represents the share (%) of the cohort out of the national count of population aged 18-64 in 2012 (201,136,985). Therefore, the overall share of the Hispanic population in the study states is obtained by the ratio between the cell figure for All study states (13.68%) and for All states (16.49%), which is 83.0%.
3) The shaded states are the non-expansion states in this study.

eMethods. Detailed Methods
State Inpatient Discharge Data Sources

Our primary data sources are state inpatient discharge databases from 17 states. These all-payer annual databases capture all discharges at all short-stay acute hospitals in the state with the exception of federally owned facilities (military and VA). Out of the 17 states, data from 12 states were obtained from Agency for Healthcare Research and Quality (AHRQ) Healthcare Cost and Utilization Project (HCUP)'s State Inpatient Databases.[1] Data from California, Illinois, Pennsylvania, Texas and Virginia were obtained directly from the respective state agencies. We used the HCUP harmonized definitions for many of the measures used in the study. Given its importance, we have detailed the definition of the race/ethnicity measure in a separate section below.

The state inpatient discharge databases are the only source of all-payer inpatient care covering the entire state population and are widely used for developing population-level national and state-level trends in inpatient healthcare utilization by the Agency for Healthcare Research and Quality (AHRQ) and other federal agencies (AHRQ 2019; Andrews 2015). They are also commonly and widely used in academic research (Smith 2011). As noted in Andrews (2015), limitations of these data include the quality of the data elements, including diagnostic and procedure codes. One source of quality issues is variation across states in the administrative agency that develops the database, which may be a state agency, state hospital association or a private data organization. The basic data instrument across the country is the Uniform Bill (UB-04), the national hospital claims standard. Differences in coding may arise at hospital and state levels. To our knowledge, this is no authoritative evidence on the accuracy of the diagnostic and procedure codes overall. There have been recent efforts at developing systematic evidence by linking the state discharge data with individual hospital entities (Pine 2015).

To our knowledge there is no evidence on the accuracy of state inpatient discharge data in identifying bariatric surgeries. Given this uncertainty, we point out two elements of our study design and analytic strategy that may limit unobserved confounding. First, by using state-level fixed effects regression models, estimates are based on intra-state variation in the outcomes; that is, systematic differences in quality of data, say, between expansion and non-expansion states, do not affect model estimates. Second, use of those aged 65-74 as within-state controls, allows capture of time-varying changes that are unrelated to Medicaid expansion. These may include technological changes in bariatric surgery types, practice patterns, and coding of administrative claims for such services.

An important issue, that we have tried to measure directly, is the change from ICD-9 to ICD-10 coding system in 2015. We find that the count the bariatric surgeries shows no discernable shift before and October 1, 2015 (see eTables 4a and 4b below).

eReferences

- Andrews, R. M. (2015). "Statewide Hospital Discharge Data: Collection, Use, Limitations, and Improvements." Health Services Research 50(S1).
- Agency for Healthcare Research and Quality (2019). 2018 National Healthcare Quality and Disparities Report. Rockville, MD, Agency for Healthcare Research & Quality. https://www.ahrq.gov/research/findings/nhqrdr/nhqdr18/index.html.
- Pine, M., N. M. Kowlessar, J. L. Salemi, J. Miyamura, D. S. Zingmond, N. E. Katz and J. Schindler (2015). "Enhancing Clinical Content and Race/Ethnicity Data in Statewide Hospital Administrative Databases: Obstacles Encountered, Strategies Adopted, and Lessons Learned." Health Serv Res 50 Suppl 1: 1300-1321.
-Smith, A. K., J. Z. Ayanian, K. E. Covinsky, B. E. Landon, E. P. McCarthy, C. C. Wee and M. A. Steinman (2011). "Conducting high-value secondary dataset analysis: an introductory guide and resources." J Gen Intern Med **26**(8): 920-929.

Identification of bariatric surgery

Our focus was on identifying primary elective bariatric surgery in the discharge data which include ICD-9-CM (2012 to 2015Q3) and ICD-10-CM / ICD-10-PCS (2015Q4-2017) codes. We did not study surgery revisions or repairs. As prior studies have differed in the choice of codes used we used two approaches. First, to be comprehensive we identified all codes used in any of the prior studies identified. Second, as use of some codes may have varied over time and across providers, we also identified counts based on the dominant single code used for laparoscopic Roux-en-Y Gastric Bypass (RYGB) gastric bypass and sleeve gastrectomy (SG) procedures across the study period (2012-2017) (these codes are shaded in the tables below). As we document below, these two codes accounted for an overwhelming share of the total volume in both the ICD-9 and ICD-10 periods. The list of prior studies we reviewed are listed below.

We identified elective primary bariatric surgeries as all discharges that meet the following conditions:

1. Use of RYGB, SG and other gastric procedure identified below.
2. Diagnosis of obesity identified as a principal or secondary diagnosis codes.
3. No diagnosis of abdominal neoplasm, ulcer or other conditions (identified below) identified in the principal or secondary diagnosis codes.
4. Patient admission was not through the emergency department. Such discharges were identified using the "Source" code in the discharge record.

eTable 2. Identification of gastric surgeries

A. During 1/1/2010 to 9/30/2015

Procedure / Condition	ICD-9 codes
Procedures (ICD-9 procedure codes)	
Roux-en-Y Gastric Bypass (RYGB)	44.38 – Laparoscopic gastroenterostomy
Vertical sleeve gastrectomy	43.82 – Laparoscopic vertical sleeve gastrectomy
Other gastric bypass procedures	43.89 – Open and other partial gastrectomy<br>44.31 - High gastric bypass<br>44.39 – Other gastroenterostomy without gastrectomy<br>44.68 - Laparoscopic gastroplasty<br>44.95 - Laparoscopic gastric restriction procedure<br>44.99 - Gastric operation nec<br>45.51 - Small bowel segment isolation<br>45.91 - Small-to-small bowel anastomosis
Diagnostic conditions (ICD-9-CM codes)	
Obesity	278.0, 278.01, 278.02 – Overweight and obesity<br>V77.8 – Screening for obesity
Abdominal conditions	150-159.9 – Abdominal neoplasms<br>230.1-230.9 – Carcinoma in situ of digestive organs<br>531.xx – 533.xx – Perforated gastrointestinal ulcer

eTable 2. Identification of gastric surgeries
B During 10/1/2015 to 12/31/2017

Procedure / Condition	ICD-10 codes
Procedures (ICD-10-PCS procedure codes)	
Roux-en-Y Gastric Bypass (RYGB)	0D164ZA- Bypass Stomach to Jejunum, Percutaneous Endoscopic Approach<br>0D1647A - Bypass Stomach to Jejunum with Autol Sub, Perc Endo Approach<br>0D1647B - Bypass Stomach to Ileum with Autol Sub, Perc Endo Approach<br>0D1687A - Bypass Stomach to Jejunum with Autol Sub, Endo<br>0D160ZA - Bypass Stomach to Jejunum, Open Approach<br>0D168ZA - Bypass Stomach to Jejunum, Endo<br>0D168ZB - Bypass stomach to ileum
Vertical sleeve gastrectomy	0DB64Z3 - Excision of Stomach, Percutaneous Endoscopic Approach, Vert<br>0DB60Z3 - Excision of Stomach, Open Approach, Vertical<br>0DB63Z3 - Excision of Stomach, Percutaneous Approach, Vertical<br>0DB67Z3 - Excision of Stomach, Via Natural or Artificial Opening, Vert<br>0DB68Z3 - Excision of Stomach, Endo, Vert
Other gastric bypass procedures	0D1607A - Bypass Stomach to Jejunum with Autol Sub, Open Approach<br>0D160ZB - Bypass Stomach to Ileum, Open Approach<br>0D164ZB - Bypass Stomach to Ileum, Percutaneous Endoscopic Approach<br>0D190ZA - Bypass Duodenum to Jejunum, Open Approach<br>0D190ZB - Bypass Duodenum to Ileum, Open Approach<br>0D194ZA - Bypass Duodenum to Jejunum, Percutaneous Endoscopic Appro.<br>0D194ZB - Bypass Duodenum to Ileum, Percutaneous Endoscopic Approach<br>0DV60CZ - Restriction of Stomach with Extralum Dev, Open Approach<br>0DV63CZ - Restriction of Stomach with Extralum Dev, Perc Approach<br>0DV64CZ - Restriction of Stomach with Extralum Dev, Perc Endo Approach
Diagnostic conditions (ICD-10-CM codes)	
Obesity	EE66.01 – Overweight and obesity
Abdominal conditions	C15.x - Malignant neoplasm of esophagus<br>C16.x - Malignant neoplasm of stomach<br>C17.x - Malignant neoplasm of small intestine<br>C25.x - Malignant neoplasm of pancreas<br>K25.x - Gastric ulcer<br>K26.x - Duodenal ulcer<br>K27.x - Peptic ulcer

eReferences:

Lewis, K. H., D. E. Arterburn, K. Callaway, F. Zhang, S. Argetsinger, J. Wallace, A. Fernandez, D. Ross-Degnan and J. F. Wharam (2019). "Risk of Operative and Nonoperative Interventions Up to 4 Years After Roux-en-Y Gastric Bypass vs Vertical Sleeve Gastrectomy in a Nationwide US Commercial Insurance Claims Database." JAMA Netw Open **2**(12): e1917603.
Khalid, S. I., P. A. Omotosho, A. Spagnoli and A. Torquati (2020). "Association of Bariatric Surgery With Risk of Fracture in Patients With Severe Obesity." JAMA Network Open **3**(6): e207419-e207419.
Ibrahim, A. M., A. A. Ghaferi, J. R. Thumma and J. B. Dimick (2017). "Hospital Quality and Medicare Expenditures for Bariatric Surgery in the United States." Ann Surg 266(1): 105-110.
Davis, M. M., K. Slish, C. Chao and M. D. Cabana (2006). "National trends in bariatric surgery, 1996-2002." Arch Surg 141(1): 71-74; discussion 75.

Identification of race/ethnicity

As discharge data are developed at the hospital level, patient race and ethnicity may not be based comprehensively on patient self-report (Hasnain-Wynia et al. 2004). As such, it is generally accepted that the quality of the race and ethnicity data is variable (Hasnain-Wynia et al. 2004; Hasnain-Wynia et al. 2006). In the analyses of the state inpatient discharge data for the development of state and national rates, the Agency for Healthcare Research and Quality (AHRQ) identifies and excludes records from hospitals with "suspect" coding of race and ethnicity (Coffey et al. 2012). Such hospitals are identified based on the following criteria:

- more than 30% of the discharges in the hospital had the race reported as “other”;
- more than 50% of the discharges had no information on the race of the patient;
- all of the discharges in the hospital had race coded as white, other, or missing;
- 100% of the discharges had race coded as white and the hospital had more than 50 beds

We followed the same procedure and excluded all bariatric surgeries from hospitals that met one of the above criteria (see eTable 3 below). As our data did not include number of hospital beds, we modified the fourth criterion and excluded hospitals with 100 percent of discharges coded as non-Hispanic White.

We used the combined categorization of race and ethnicity into the 5 groups identified in Table 1. This categorization was developed by AHRQ and the combined categorization field was included in the raw data we obtained.

eReferences:

Coffey, R. M., M. Barrett, R. Houchens, E. Moy, R. Andrews, E. Moles and N. Coenen (2012). Methods Applying AHRQ Quality Indicators to Healthcare Cost and Utilization Project (HCUP) Data for the Tenth (2012) National Healthcare Quality Report (NHQR) and National Healthcare Disparities Report (NHDR), U.S. Agency for Healthcare Research and Quality.

Hasnain-Wynia, R., D. Pierce and M. Pittman (2004). Who, when, and how: The current state of race, ethnicity, and primary language data collection in hospitals, Commonwealth Fund New York.

Hasnain-Wynia, R. and D. W. Baker (2006). "Obtaining Data on Patient Race, Ethnicity, and Primary Language in Health Care Organizations: Current Challenges and Proposed Solutions." Health Services Research **41**(4p1): 1501-1518.

eTable 3. Cohorts and Counts of Bariatric Surgeries

eTable 3a. Study cohort of bariatric surgeries, 2010 to 2017

Exclusion condition	# excluded cases (%)	# cases
Initial surgery count, age 26-74		686,278
Patient not resident of state where surgery performed	28,349	657,929
Hospital admission through the emergency department	6,232	651,697
Patient sex missing	73	651,624
Suspect race/ethnicity	14,067	637,557
Total		637,557

Notes:
1) The states included in the study are 11 expansion states: Arkansas, Arizona, California, Colorado, Iowa, Illinois, Kentucky, New Jersey, New York, Oregon and Pennsylvania 6 non-expansion states: Florida, Georgia, North Carolina, Texas, Virginia and Wisconsin. Data for all years (2010-2017) were not available for all the 17 study states. Specifically, we did not have 2010-2011 data for WI and 2017 data for AR and NY.
2) The initial surgery count represents all the bariatric surgeries identified for patients aged 26-74. Bariatric surgeries were identified using ICD-9 and ICD-10 procedure and diagnosis codes. Details are in eTable 4.

eTable 3b. Counts of bariatric surgeries (ICD-9 procedure codes), 1/1/2010 – 9/30/2015

ICD-9 Procedure Code	Procedure name	Counts of surgery by year								
		2010	2011	2012	2013	2014	2015Q1	2015Q2	2015Q3	2015Q1-Q3
4438	Laparoscopic gastroenterostomy	37,860	34,720	33,663	29,839	27,334	5,907	6,570	6,426	18,903
4382	Gastroenterostomy nec	0	5,478	30,482	44,980	55,247	13,596	15,131	15,706	44,433
4382 / 4438	Indeterminate (both codes identified)	0	4	196	141	119	19	27	22	68
4389	Open and other partial gastrectomy	8,904	13,458	1,042	1,024	1,026	268	364	376	1,008
4431	High gastric bypass	251	213	158	100	82	25	25	20	70
4439	Other gastroenterostomy without gastrectomy	2633	2204	1,813	1,224	1,127	259	271	301	831
4468	Laparoscopic gastroplasty	501	367	346	270	187	15	15	22	52
4495	Laparoscopic gastric restriction procedure	13715	8786	4,059	1,625	759	137	100	101	338
4499	Other operation on stomach	120	120	94	97	82	16	16	21	53
4551	Small bowel segment isolation	30	59	35	43	52	11	15	6	32
4591	Small-to-small bowel anastomosis	678	613	364	319	312	93	117	100	310
Total		64,692	66,022	72,252	79,662	86,327	20,346	22,651	23,101	66,098

Notes:
1) These counts do not include the cases excluded for the criteria identified in eTable 3.
2) We have broken down the counts in 2015 by individual quarter (the three quarters denoted by Q1, Q2 and Q3), to assist in reviewing shifts in volume that may have occurred after the switch from ICD-9 to ICD-10 codes.
3) Note the shift in surgery type longitudinally. The main bypass procedure (ICD9 = 4438) and laparoscopic procedure (ICD9=4382) accounted for 84.5% of all surgeries during 2010 to 2015Q3. However, in 2010, the predominant laparoscopic procedure was 4495, and was largely replaced by 4382 in the subsequent three years. In 2015Q1-Q3, 4438 and 4382 accounted for 97.9 percent of all surgeries. During 2015Q4 to 2017, after switch to ICD10, the corresponding two procedures (0D164ZA and 0DB64Z3) accounted for 97.5 percent of all surgeries.

eTable 3c. Counts of bariatric surgeries (ICD-10 procedure codes), 10/1/2015 – 12/31/2017

ICD-10 Procedure Code	Procedure name	Counts of surgery by year		
		2015q4	2016	2017
0D164ZA	Bypass Stomach to Jejunum, Percutaneous Endoscopic Approach	6,504	24,386	20,024
0DB64Z3	Excision of Stomach, Percutaneous Endoscopic Approach, Vert	16,881	69,187	62,090
0D164ZA	Indeterminat (both codes above identified)	35	139	122
	Other RYGB surgeries			
0D1647A	Bypass Stomach to Jejunum with Autol Sub, Perc Endo Approach	110	266	241
0D1647B	Bypass Stomach to Ileum with Autol Sub, Perc Endo Approach	0	3	2
0D1687A	Bypass Stomach to Jejunum with Autol Sub, Endo	0	1	5
0D160ZA	Bypass Stomach to Jejunum, Open Approach	202	688	553
0D168ZA	Bypass Stomach to Jejunum, Endo	42	84	74
0D168ZB	bypass stomach to ileum	0	0	2
	Other VSG surgeries			
0DB60Z3	Excision of Stomach, Open Approach, Vertical	155	480	476
0DB63Z3	Excision of Stomach, Percutaneous Approach, Vertical	43	58	5
0DB67Z3	Excision of Stomach, Via Natural or Artificial Opening, Vert	5	6	1
0DB68Z3	Excision of Stomach, Endo, Vert	72	128	16
	Other bariatric procedures			
0D1607A	Bypass Stomach to Jejunum with Autol Sub, Open Approach	5	10	3
0D160ZB	Bypass Stomach to Ileum, Open Approach	1	12	16
0D164ZB	Bypass Stomach to Ileum, Percutaneous Endoscopic Approach			
0D190ZA	Bypass Duodenum to Jejunum, Open Approach	0	5	6
0D190ZB	Bypass Duodenum to Ileum, Open Approach	1	16	37
0D194ZA	Bypass Duodenum to Jejunum, Percutaneous Endoscopic Approach	1	0	0
0D194ZB	Bypass Duodenum to Ileum, Percutaneous Endoscopic Approach	0	0	4
0DV60CZ	Restriction of Stomach with Extralum Dev, Open Approach	5	6	5
0DV63CZ	Restriction of Stomach with Extralum Dev, Perc Approach	2	2	0
0DV64CZ	Restriction of Stomach with Extralum Dev, Perc Endo Approach	209	453	229
	Total	24,273	95,930	83,911

Notes:
1) These counts do not include the cases excluded for the criteria in eTable 3.
2) We find no noticeable change in surgery volume associated with shift from ICD9 to ICD10.The volume in the last ICD-9 quarter (2015Q3) was 23,101 and in the first ICD-10 quarter (2015Q4) was 24,273. This magnitude of change may also be due to (a) secular longitudinal increase in procedure volume, and (b) seasonal differences.
3) The first two procedure codes (0D164ZA, 0DB64Z3) account for 97.5 percent of the total volume during this period.

Estimation data and models:

The analytic data is longitudinal state-cohort level data for each of the three outcomes of interest: number of bariatric surgeries, count of census population and the rate of bariatric surgery (per 10,000 census population). Each regression was based on 264 observations, consisting of 17 states x (up to) 8 years x 2 age groups (26-64 and 65-74). As noted in Table 1, we had 8 years of data for all states, except WI (6 years) and AR and NY (7 years).

Our preferred regression was a log-linear regression model using an event study specification of difference in differences. We preferred an event study specification over the canonical (pre/post) specification because (a) not all states expanded Medicaid in the same year, and (b) the potential impact of expansion may vary over time. (Ryan et al 2015; Wing et al 2018)). Also, we used a three-way difference in differences specification based on comparison with two controls: non-expansion states, and (within state) older age group (65-74). Use of in-state controls not directly affected by expansion allows the capture of changes in bariatric surgery not affected by expansion (e.g., changes in the technology of bariatric surgery and practice patterns in its utilization).

We defined two dichotomous indicators of the two control groups: expansion status (1 = expansion state; 0 = non-expansion state) and age group (1 = age 26-64 and 0 = age 65-74). To estimate the change in the number of surgeries in each calendar year, we defined dichotomous indicators of each calendar year (2010 to 2017) relative to the date of expansion. Medicaid expansion occurred on 1/1/2014 in ten of the expansion states and on 1/1/2015 in Pennsylvania. So, indicator of the first year after expansion was defined as value 1 for year 2014 in all states other than PA and for year 2015 in PA, and as value 0 for other years. Similarly, we defined indicators of 2, 3 and 4 years after expansion, and 1 to 3 years before expansion (for PA both 2012 and 2013 were grouped as 2 years before expansion). In the regression model we included, as covariates, the above mentioned relative year indicators (with one year prior to expansion as the reference period), indicators (0/1) of state expansion status and age group, and all two-way and three-way interaction of the three sets of indicators.

$$E(y_{st}) = \beta_{1t} * age2664_{st} + \beta_{2t} * expansion_s + \beta_{3t} * relyear_t + \beta_{4t} * age2664_{st} * relyear_t + \beta_{5t} * expansion_s * relyear_t + \beta_{6t} * age2664_{st} * expansion_s * relyear_t + I.state \quad \text{(Model 1)}$$

y_{st} is the outcome measure in state s in year t. $relyear_t$ denotes the year indicator relative to the base year described above. We included fixed effects indicators for each state. Inclusion of state fixed effects adjusts for the unobserved time-invariant heterogeneity across areas by utilizing only the within-state changes in surgery counts; specifically, the estimates are robust to state-level differences in time-invariant characteristics, such as the sociodemographic composition, local healthcare environment (including provider availability) and rural location. We obtained standard errors clustered at the state level (Hansen 2020).

Our preferred specification was a log-linear model wherein we used the logarithm of the outcome measure (y_{st}). This enables interpretation of the interaction estimates as percentage change in outcome in the relative year ($relyear_t$) relative to that in the base year, associated with Medicaid expansion. Included in the relative years is the comparison of the three pre-expansion years (2010 to 2013). This estimate is an important indicator of the validity of the non-expansion states as a suitable comparator ("parallel trends test");[2] since these two years precede Medicaid expansion, a significant non-zero estimate weakens the case for validity of the comparison group of state as it indicates the presence of systematic difference in the longitudinal trend in outcome measure prior to expansion.

This three-way difference-in-differences regression model is estimated separately for each outcome for a variety of subgroups (insurance coverage, race/ethnicity, age and sex).

Alongside the above regression model, in each case we also estimated a modified version of the relative year wherein all the years following expansion are combined, to give the average change in surgery volume during the 2014-2017 period (relative to the reference year); this is denoted as $relyear2_t$.

$$E(y_{st}) = \beta_{1t} * age2664_{st} + \beta_{2t} * expansion_s + \beta_{3t} * relyear2_t + \beta_{4t} * age2664_{st} * relyear2_t + \beta_{5t} * expansion_s * relyear2_t + \beta_{6t} * age2664_{st} * expansion_s * relyear2_t + I.state$$

(Model 2)

As sensitivity analysis we also estimated the Poisson specification of the above linear models.

eReferences:

Cunningham, S. (2021). Causal Inference, Yale University Press.

Garthwaite, C., J. A. Graves, T. Gross, Z. Karaca, V. R. Marone and M. J. Notowidigdo (2019). "All Medicaid Expansions Are Not Created Equal: The Geography and Targeting of the Affordable Care Act." National Bureau of Economic Research Working Paper Series No. 26289.

Hansen BE. Econometrics. https://www.ssc.wisc.edu/~bhansen/econometrics/2020.
Health Resources & Services Administration (2020). Defining Rural Population. https://www.hrsa.gov/rural-health/about-us/definition/index.html.

Ryan AM, Burgess JF, Jr., Dimick JB. Why We Should Not Be Indifferent to Specification Choices for Difference-in-Differences. Health Serv Res. 2015;50(4):1211-1235.

Venkataramani, A. S., E. F. Bair, R. L. O'Brien and A. C. Tsai (2020). "Association Between Automotive Assembly Plant Closures and Opioid Overdose Mortality in the United States: A Difference-in-Differences Analysis." JAMA Internal Medicine 180(2): 254-262.

Wing C, Simon K, Bello-Gomez RA. Designing Difference in Difference Studies: Best Practices for Public Health Policy Research. Annual Review of Public Health. 2018;39(1):453-469.

eFigure 2. Longitudinal volume of bariatric surgeries by insurance payer and state Medicaid expansion status

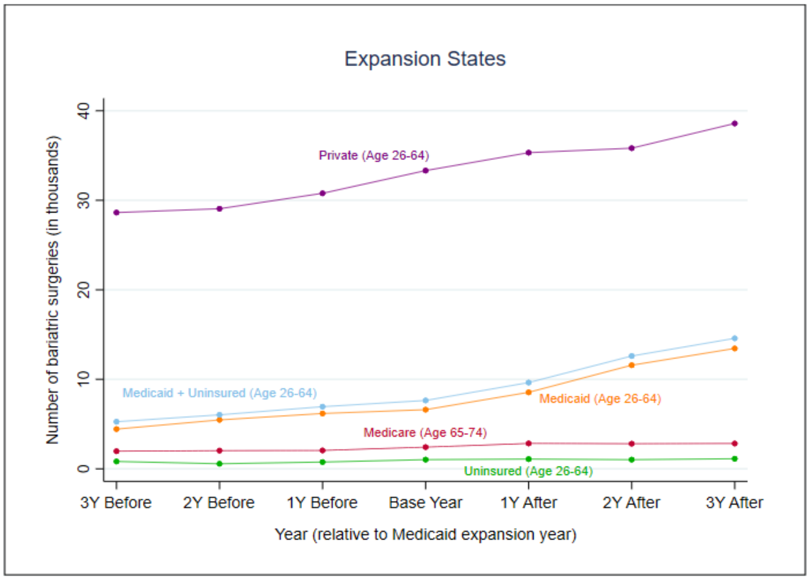

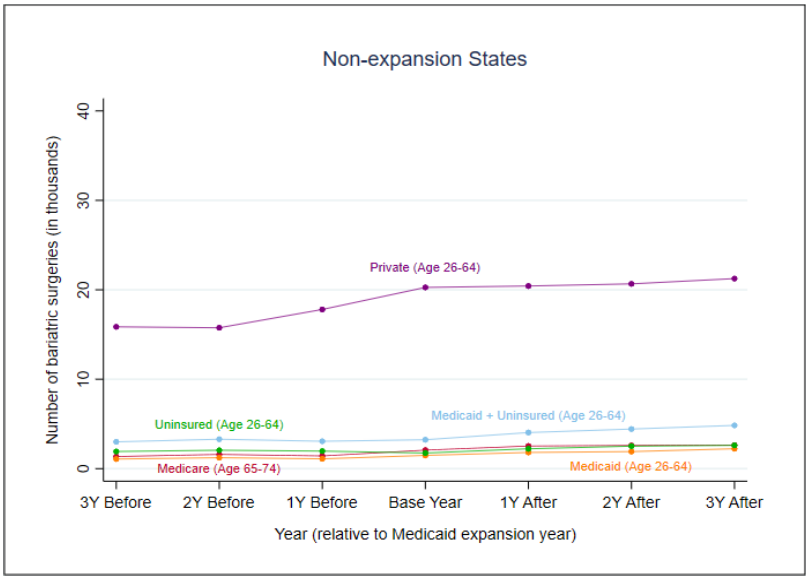

Notes:
1) Observed count of bariatric surgeries by insurance coverage from each state were aggregated for Medicaid expansion and non-expansion states separately.
2) For each state, base year is defined as either 2014 (Pennsylvania) or 2013 (other 16 states). Other years are defined relative to the base year for each state. Data for all years (2010-2017) was not available for all the 17 study states. Specifically, we lacked 2010-2011 data for WI and 2017 data for AR and NY. As a result, we have 3-year before and 3-year after base year for all states except WI. The above graphs are reported for the 16 study states other than WI.

eTable 4. Number and Changes in Bariatric Surgeries

eTable 4a1. Number of bariatric procedures by relative year

Insurance payer	3 year before base year	2 year before base year	1 year before base year	Base year (year before expansion)	Year 1 after expansion	Year 2 after expansion	Year 3 after expansion
	Expansion states						
Medicaid & uninsured	5,263	6,034	6,944	7,638	9,637	12,604	14,579
Medicaid	4,439	5,465	6,184	6,609	8,547	11,578	13,448
Uninsured	824	569	760	1,029	1,090	1,026	1,131
Private	28,629	29,056	30,779	33,319	35,318	35,821	38,578
Others	6,725	6,677	6,577	7,764	8,772	8,842	8,870
Age 26-64	4,757	4,641	4,523	5,338	5,935	6,034	6,039
Age 65-74	1,968	2,036	2,054	2,426	2,837	2,808	2,831
All payers (age 26-64)	38,649	39,731	42,246	46,295	50,890	54,459	59,196
	Non-expansion states						
Medicaid & uninsured	3,003	3,294	3,066	3,237	4,040	4,432	4,839
Medicaid	1,081	1,229	1,101	1,486	1,814	1,911	2,229
Uninsured	1,922	2,065	1,965	1,751	2,226	2,521	2,610
Private	15,860	15,755	17,802	20,267	20,425	20,665	21,252
Others	4,782	5,251	5,951	6,019	6,844	7,024	7,164
Age 26-64	3,421	3,658	4,525	3,939	4,309	4,423	4,526
Age 65-74	1,361	1,593	1,426	2,080	2,535	2,601	2,638
All payers (age 26-64)	22,284	22,707	25,393	27,443	28,774	29,520	30,617

eTable 4a2. Longitudinal change in number of bariatric procedures: Base year = 100

Insurance payer	3 year before base year	2 year before base year	1 year before base year	Base year (year before expansion)	Year 1 after expansion	Year 2 after expansion	Year 3 after expansion
	Expansion states						
Medicaid & uninsured	68.9	79.0	90.9	100	126.2	165.0	190.9
Medicaid	67.2	82.7	93.6	100	129.3	175.2	203.5
Uninsured	80.1	55.3	73.9	100	105.9	99.7	109.9
Private	85.9	87.2	92.4	100	106.0	107.5	115.8
Others	86.6	86.0	84.7	100	113.0	113.9	114.2
Age 26-64	89.1	86.9	84.7	100	111.2	113.0	113.1
Age 65-74	81.1	83.9	84.7	100	116.9	115.7	116.7
All payers (age 26-64)	83.5	85.8	91.3	100	109.9	117.6	127.9
	Non-expansion states						
Medicaid & uninsured	92.8	101.8	94.7	100	124.8	136.9	149.5
Medicaid	72.7	82.7	74.1	100	122.1	128.6	150.0
Uninsured	109.8	117.9	112.2	100	127.1	144.0	149.1
Private	78.3	77.7	87.8	100	100.8	102.0	104.9
Others	79.4	87.2	98.9	100	113.7	116.7	119.0
Age 26-64	86.8	92.9	114.9	100	109.4	112.3	114.9
Age 65-74	65.4	76.6	68.6	100	121.9	125.0	126.8
All payers (age 26-64)	81.2	82.7	92.5	100	104.9	107.6	111.6

Notes:
1) Observed count of bariatric surgeries by insurance coverage from each state were aggregated for Medicaid expansion and non-expansion states separately.
2) For each state, base year is defined as either 2014 (Pennsylvania) or 2013 (other 16 states). Other years are defined relative to the base year for each state. Data for all years (2010-2017) was not available for all the 17 study states. Specifically, we lacked 2010-2011 data for WI and 2017 data for AR and NY. As a result, we have 3-year before and 3-year after base year for all states except WI. The above graphs are reported for the 16 study states other than WI.
3) Longitudinal change in measured as a ratio between the count for each year (for each payer) with the base year count (for the same payer), setting the base year value to 100. So, a ratio of 110.3 denotes as 10.3 percent increase. Correspondingly, ratio < 100 denotes lower than baseline count.

eTable 4b. Change (%) in volume of bariatric surgeries associated with Medicaid expansion: Full model estimates

Regression covariate	Medicaid + Uninsured	Medicaid	Uninsured	Private	All payer groups
Age 26-64 (reference: age 65+)	57.5 [8.8, 127.9]	-26.3 [-64.6, 53.3]	-29.0 [-52.5, 6.1]	845.8 [460.8, 1495.2]	1264.4 [830.8, 1899.9]
Age 26-64 x Medicaid expansion	85.1 [6.4, 222.0]	223.8 [35.3, 675.1]	-46.1 [-68.9, -6.6]	47.9 [-17.3, 164.8]	42.4 [-8.8, 122.4]
Year 3 before base year	-35.8 [-48.1, -20.5]	-34.1 [-47.2, -17.7]	-38.9 [-51.6, -22.9]	-37.2 [-50.2, -20.7]	-36.9 [-49.7, -20.9]
Year 2 before base year	-21.0 [-39.1, 2.5]	-18.9 [-37.7, 5.7]	-24.9 [-42.8, -1.3]	-22.7 [-40.4, 0.2]	-22.4 [-39.9, 0.2]
Year 1 before base year	-27.8 [-43.2, -8.0]	-27.8 [-43.2, -8.0]	-27.8 [-43.2, -8.0]	-27.8 [-43.2, -8.0]	-27.8 [-43.2, -8.0]
Year 1 after base year	22.8 [15.7, 30.4]	22.8 [15.7, 30.4]	22.8 [15.7, 30.4]	22.8 [15.7, 30.4]	22.8 [15.7, 30.4]
Year 2 after base year	28.6 [17.1, 41.3]	28.6 [17.1, 41.3]	28.6 [17.1, 41.3]	28.6 [17.1, 41.3]	28.6 [17.1, 41.3]
Year 3 after base year	26.1 [14.6, 38.7]	26.1 [14.6, 38.7]	26.1 [14.6, 38.7]	26.1 [14.6, 38.7]	26.1 [14.6, 38.7]
Year 4 after base year	28.5 [21.2, 36.2]	28.5 [21.2, 36.2]	28.5 [21.2, 36.2]	28.5 [21.2, 36.2]	28.5 [21.2, 36.2]
Age 26-64 x Year 3 before base year	35.4 [-17.8, 123.1]	15.7 [-39.5, 121.4]	53.8 [0.9, 134.5]	27.0 [1.5, 58.9]	28.0 [3.5, 58.2]
Age 26-64 x Year 2 before base year	8.3 [-48.0, 125.6]	-4.4 [-59.0, 122.9]	29.6 [-31.1, 144.0]	2.3 [-20.4, 31.4]	6.1 [-17.7, 36.9]
Age 26-64 x Year 1 before base year	14.6 [-41.1, 122.9]	-3.4 [-48.9, 82.5]	24.9 [-32.4, 130.6]	21.1 [-6.0, 56.0]	25.7 [-8.9, 73.3]
Age 26-64 x Year 1 after base year	-5.8 [-19.7, 10.4]	-13.1 [-33.0, 12.8]	-10.0 [-38.8, 32.4]	-16.0 [-27.4, -2.9]	-14.2 [-24.4, -2.7]
Age 26-64 x Year 2 after base year	0.1 [-14.4, 17.2]	-7.0 [-21.6, 10.4]	-2.9 [-30.6, 35.9]	-18.5 [-32.8, -1.1]	-15.9 [-29.0, -0.3]
Age 26-64 x Year 3 after base year	14.7 [0.6, 30.8]	14.1 [-10.1, 44.7]	2.3 [-24.9, 39.4]	-14.9 [-32.4, 7.2]	-11.6 [-27.9, 8.3]
Age 26-64 x Year 4 after base year	20.7 [-0.5, 46.5]	27.6 [0.3, 62.4]	-3.3 [-40.6, 57.4]	-17.4 [-37.1, 8.4]	-12.8 [-31.1, 10.3]
Medicaid expansion x Year 3 before base year	27.4 [-14.1, 89.0]	24.1 [-16.7, 84.8]	34.0 [-10.7, 101.0]	30.2 [-13.2, 95.3]	29.7 [-13.2, 93.9]
Medicaid expansion x Year 2 before base year	8.4 [-30.4, 69.0]	5.6 [-32.4, 64.9]	14.0 [-27.4, 79.1]	10.8 [-28.9, 72.6]	10.4 [-29.0, 71.6]
Medicaid expansion x Year 1 before base year	19.3 [-14.5, 66.6]	19.3 [-14.5, 66.6]	19.3 [-14.5, 66.6]	19.3 [-14.5, 66.6]	19.3 [-14.5, 66.6]
Medicaid expansion x Year 1 after base year	-4.3 [-15.6, 8.4]	-4.3 [-15.6, 8.4]	-4.3 [-15.6, 8.4]	-4.3 [-15.6, 8.4]	-4.3 [-15.6, 8.4]
Medicaid expansion x Year 2 after base year	-9.0 [-21.3, 5.1]	-9.0 [-21.3, 5.1]	-9.0 [-21.3, 5.1]	-9.0 [-21.3, 5.1]	-9.0 [-21.3, 5.1]
Medicaid expansion x Year 3 after base year	-5.2 [-18.5, 10.3]	-5.2 [-18.5, 10.3]	-5.2 [-18.5, 10.3]	-5.2 [-18.5, 10.3]	-5.2 [-18.5, 10.3]
Medicaid expansion x Year 4 after base year	-8.5 [-37.8, 34.7]	-6.8 [-36.4, 36.5]	-19.0 [-47.5, 25.2]	-16.2 [-42.3, 21.8]	-14.9 [-41.5, 23.7]
Age 26-64 x Medicaid expansion x Year 3 before base year	-37.8 [-67.4, 18.7]	-29.8 [-69.2, 59.6]	-33.1 [-59.3, 9.9]	-18.1 [-38.7, 9.5]	-19.3 [-38.8, 6.4]
Age 26-64 x Medicaid expansion x Year 2 before base year	-14.2 [-63.7, 102.9]	2.8 [-62.2, 179.3]	-43.6 [-72.1, 13.7]	-3.1 [-29.8, 33.8]	-5.0 [-30.4, 29.6]

Age 26-64 x Medicaid expansion x Year 1 before base year	-5.6 [-53.3, 90.9]	16.1 [-41.7, 131.2]	-28.1 [-62.0, 36.3]	-11.1 [-32.8, 17.6]	-14.7 [-39.6, 20.3]
Age 26-64 x Medicaid expansion x Year 1 after base year	13.4 [-7.3, 38.8]	29.7 [-3.1, 73.5]	1.3 [-35.6, 59.4]	6.6 [-10.1, 26.5]	8.2 [-7.2, 26.3]
Age 26-64 x Medicaid expansion x Year 2 after base year	42.8 [10.6, 84.3]	71.2 [28.9, 127.5]	-17.0 [-46.8, 29.4]	10.9 [-11.7, 39.2]	19.0 [-2.1, 44.6]
Age 26-64 x Medicaid expansion x Year 3 after base year	43.8 [9.3, 89.3]	60.7 [11.4, 132.0]	-15.9 [-45.4, 29.7]	12.0 [-13.2, 44.5]	20.2 [-4.2, 50.8]
Age 26-64 x Medicaid expansion x Year 4 after base year	21.9 [-29.1, 109.6]	29.6 [-28.1, 133.5]	-9.1 [-52.8, 75.3]	0.4 [-30.6, 45.2]	2.9 [-30.8, 53.0]

Notes:
1) The estimates in each column are from a separate regression using observations for the respective payer group/s.
2) Estimates reported here from the log-linear regression model specification.
3) The estimate for each covariate is obtained as 100*[exp(coefficient) - 1].
4) Confidence intervals were obtained based on standard errors clustered at the state level. Estimates in bold indicate significance at p<0.05 level.

eFigure 3. Longitudinal volume of census population by insurance payer and state Medicaid expansion status

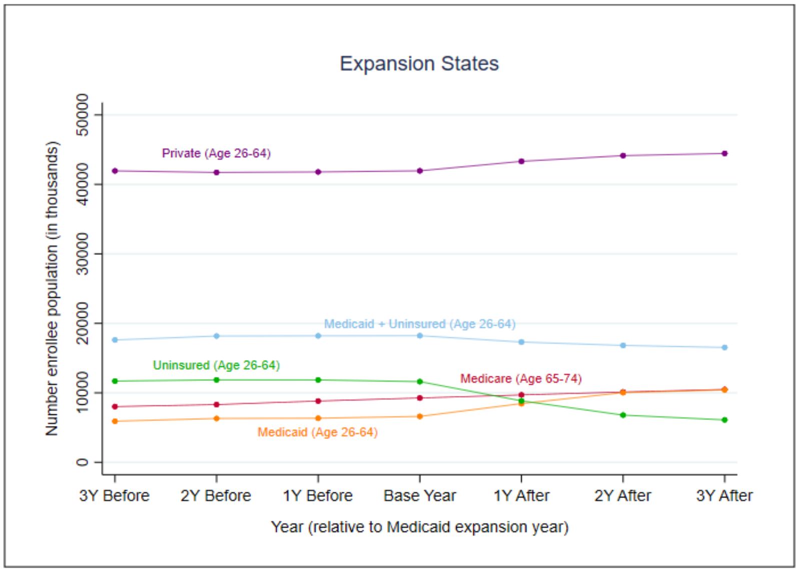

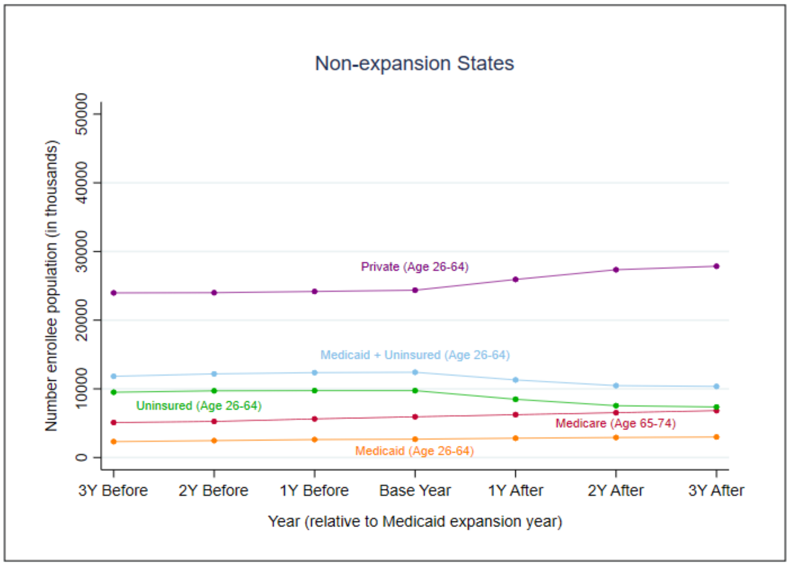

Notes:
1) Observed count of census population by insurance coverage from each state were aggregated for Medicaid expansion and non-expansion states separately.
2) We used annual census population estimates by state-payer cohorts (from the American Community Survey) for the same states and years as in eFigure 2.

eTable 5. Number and Changes in Census Population

eTable 5a1. Number of census population by relative year

Insurance payer	3 year before base year	2 year before base year	1 year before base year	Base year (year before expansion)	Year 1 after expansion	Year 2 after expansion	Year 3 after expansion
	Expansion states						
Medicaid & uninsured	17.6	18.2	18.2	18.2	17.3	16.8	16.5
Medicaid	5.9	6.3	6.3	6.6	8.5	10.0	10.4
Uninsured	11.7	11.9	11.9	11.6	8.9	6.8	6.1
Private	41.9	41.7	41.8	41.9	43.3	44.1	44.5
Others	9.0	9.3	9.9	10.4	10.8	11.2	11.5
Age 26-64	1.0	1.0	1.0	1.1	1.1	1.1	1.1
Age 65-74	8.0	8.3	8.8	9.3	9.7	10.1	10.5
All payers (age 26-64)	60.5	60.9	61.0	61.3	61.7	62.0	62.0
	Non-expansion states						
Medicaid & uninsured	11.8	12.2	12.4	12.4	11.3	10.5	10.4
Medicaid	2.3	2.5	2.6	2.7	2.8	2.9	3.0
Uninsured	9.5	9.7	9.7	9.7	8.5	7.6	7.4
Private	24.0	24.0	24.2	24.4	25.9	27.3	27.9
Others	5.9	6.1	6.5	6.8	7.2	7.4	7.7
Age 26-64	0.8	0.8	0.8	0.9	0.9	0.9	0.9
Age 65-74	5.1	5.3	5.6	5.9	6.2	6.5	6.8
All payers (age 26-64)	36.6	37.0	37.4	37.6	38.1	38.7	39.1

eTable 5a2. Longitudinal change in census population: Base year = 100

Insurance payer	3 year before base year	2 year before base year	1 year before base year	Base year (year before expansion)	Year 1 after expansion	Year 2 after expansion	Year 3 after expansion
	Expansion states						
Medicaid & uninsured	96.6	99.7	99.9	100	95.0	92.3	90.7
Medicaid	89.5	95.5	96.0	100	127.9	151.7	157.7
Uninsured	100.7	102.2	102.1	100	76.2	58.5	52.6
Private	100.0	99.5	99.6	100	103.3	105.2	106.0
Others	86.8	89.7	95.2	100	104.1	108.1	111.5
Age 26-64	87.9	89.5	94.7	100	98.8	97.6	96.4
Age 65-74	86.6	89.7	95.2	100	104.8	109.3	113.2
All payers (age 26-64)	98.8	99.4	99.6	100	100.7	101.2	101.3
	Non-expansion states						
Medicaid & uninsured	95.3	98.1	99.6	100	91.0	84.3	83.4
Medicaid	86.8	92.3	98.1	100	105.5	109.2	112.0
Uninsured	97.6	99.7	100.0	100	87.1	77.5	75.6
Private	98.4	98.5	99.2	100	106.4	112.2	114.3
Others	86.6	89.5	95.3	100	105.3	109.2	113.9
Age 26-64	91.1	95.8	98.7	100	106.0	103.5	106.2
Age 65-74	85.9	88.6	94.9	100	105.2	110.0	115.1
All payers (age 26-64)	97.2	98.3	99.3	100	101.3	102.8	103.9

Notes:
1) Observed count of bariatric surgeries by insurance coverage from each state were aggregated for Medicaid expansion and non-expansion states separately.
2) For each state, base year is defined as either 2014 (Pennsylvania) or 2013 (other 16 states). Other years are defined relative to the base year for each state. Data for all years (2010-2017) was not available for all the 17 study states. Specifically, we lacked 2010-2011 data for WI and 2017 data for AR and NY. As a result, we have 3-year before and 3-year after base year for all states except WI. The above graphs are reported for the 16 study states other than WI.
3) Longitudinal change in measured as a ratio between the count for each year (for each payer) with the base year count (for the same payer), setting the base year value to 100. So, a ratio of 110.3 denotes as 10.3 percent increase. Correspondingly, ratio < 100 denotes lower than baseline count.

eTable 5b. Change (%) in census population associated with Medicaid expansion: Full model estimates

Regression covariate	Medicaid + Uninsured	Medicaid	Uninsured	Private	All payer groups
Age 26-64 (reference: age 65+)	103.7 [60.1, 159.3]	-53.5 [-58.4, -48.1]	54.9 [13.7, 111.1]	318.3 [232.6, 426.2]	540.9 [423.4, 684.7]
Age 26-64 x Medicaid expansion	-4.3 [-31.0, 32.5]	50.9 [19.4, 90.6]	-20.9 [-48.2, 21.0]	8.4 [-14.4, 37.2]	3.5 [-17.0, 29.0]
Year 3 before base year	-15.4 [-18.8, -11.8]	-13.0 [-16.3, -9.5]	-16.9 [-23.4, -9.9]	-13.9 [-16.3, -11.5]	-14.3 [-16.5, -12.1]
Year 2 before base year	-12.7 [-15.9, -9.4]	-10.2 [-13.5, -6.9]	-14.3 [-20.8, -7.3]	-11.2 [-13.1, -9.2]	-11.6 [-13.2, -9.9]
Year 1 before base year	-5.2 [-6.1, -4.4]	-5.2 [-6.1, -4.4]	-5.2 [-6.1, -4.4]	-5.2 [-6.1, -4.4]	-5.2 [-6.1, -4.4]
Year 1 after base year	5.2 [4.7, 5.7]	5.2 [4.7, 5.7]	5.2 [4.7, 5.7]	5.2 [4.7, 5.7]	5.2 [4.7, 5.7]
Year 2 after base year	10.0 [9.2, 10.9]	10.0 [9.2, 10.9]	10.0 [9.2, 10.9]	10.0 [9.2, 10.9]	10.0 [9.2, 10.9]
Year 3 after base year	15.0 [14.3, 15.8]	15.0 [14.3, 15.8]	15.0 [14.3, 15.8]	15.0 [14.3, 15.8]	15.0 [14.3, 15.8]
Year 4 after base year	19.1 [18.3, 19.9]	19.1 [18.3, 19.9]	19.1 [18.3, 19.9]	19.1 [18.3, 19.9]	19.1 [18.3, 19.9]
Age 26-64 x Year 3 before base year	13.9 [5.4, 23.2]	-1.3 [-12.9, 11.9]	19.9 [5.0, 37.0]	13.4 [9.0, 18.0]	13.4 [9.8, 17.1]
Age 26-64 x Year 2 before base year	13.6 [5.5, 22.5]	1.6 [-8.5, 12.8]	18.7 [4.0, 35.6]	10.1 [6.5, 13.7]	11.1 [8.4, 13.9]
Age 26-64 x Year 1 before base year	5.3 [3.0, 7.6]	3.5 [0.3, 6.9]	5.9 [3.4, 8.4]	4.7 [3.5, 5.9]	4.9 [3.9, 5.9]
Age 26-64 x Year 1 after base year	-13.6 [-14.4, -12.8]	-0.1 [-2.3, 2.2]	-17.9 [-20.4, -15.5]	1.0 [-0.5, 2.6]	-3.8 [-4.4, -3.2]
Age 26-64 x Year 2 after base year	-23.3 [-25.4, -21.3]	-1.3 [-6.7, 4.3]	-30.7 [-35.5, -25.5]	1.7 [-1.4, 4.9]	-6.7 [-7.4, -6.1]
Age 26-64 x Year 3 after base year	-27.7 [-30.2, -25.1]	-3.5 [-8.8, 2.2]	-36.0 [-42.1, -29.3]	-1.0 [-4.7, 2.8]	-10.0 [-11.4, -8.5]
Age 26-64 x Year 4 after base year	-28.3 [-31.5, -25.0]	-7.0 [-10.6, -3.2]	-35.7 [-42.2, -28.5]	-3.5 [-7.9, 1.1]	-11.8 [-13.8, -9.8]
Medicaid expansion x Year 3 before base year	2.3 [-2.3, 7.1]	-0.5 [-4.8, 4.0]	4.2 [-4.2, 13.3]	0.5 [-2.9, 4.0]	1.0 [-2.2, 4.4]
Medicaid expansion x Year 2 before base year	2.7 [-1.4, 7.0]	-0.1 [-4.1, 4.0]	4.6 [-3.5, 13.4]	0.9 [-1.8, 3.7]	1.4 [-1.1, 4.0]
Medicaid expansion x Year 1 before base year	0.4 [-1.1, 1.9]	0.4 [-1.1, 1.9]	0.4 [-1.1, 1.9]	0.4 [-1.1, 1.9]	0.4 [-1.1, 1.9]
Medicaid expansion x Year 1 after base year	-0.4 [-1.3, 0.5]	-0.4 [-1.3, 0.5]	-0.4 [-1.3, 0.5]	-0.4 [-1.3, 0.5]	-0.4 [-1.3, 0.5]
Medicaid expansion x Year 2 after base year	-0.6 [-2.1, 0.9]	-0.6 [-2.1, 0.9]	-0.6 [-2.1, 0.9]	-0.6 [-2.1, 0.9]	-0.6 [-2.1, 0.9]
Medicaid expansion x Year 3 after base year	-1.6 [-3.2, 0.1]	-1.6 [-3.2, 0.1]	-1.6 [-3.2, 0.1]	-1.6 [-3.2, 0.1]	-1.6 [-3.2, 0.1]
Medicaid expansion x Year 4 after base year	-3.3 [-8.7, 2.3]	0.4 [-6.9, 8.3]	-6.1 [-13.2, 1.5]	-1.4 [-3.5, 0.8]	-2.0 [-4.5, 0.7]
Age 26-64 x Medicaid expansion x Year 3 before base year	-1.7 [-9.8, 7.1]	4.8 [-8.4, 19.8]	-2.4 [-15.1, 12.2]	1.8 [-2.3, 6.2]	0.7 [-2.8, 4.2]
Age 26-64 x Medicaid expansion x Year 2 before base year	-2.0 [-9.2, 5.8]	4.7 [-6.0, 16.8]	-3.7 [-15.9, 10.3]	0.8 [-2.8, 4.5]	-0.3 [-3.0, 2.5]
Age 26-64 x Medicaid expansion x Year 1 before base year	-0.2 [-3.2, 3.0]	-2.3 [-6.5, 2.0]	1.6 [-2.4, 5.8]	0.0 [-1.6, 1.5]	-0.2 [-1.5, 1.2]

Age 26-64 x Medicaid expansion x Year 1 after base year	4.8 [2.8, 6.8]	22.7 [13.4, 32.8]	-11.4 [-16.0, -6.4]	-2.4 [-4.3, -0.5]	-0.1 [-0.9, 0.7]
Age 26-64 x Medicaid expansion x Year 2 after base year	10.2 [5.3, 15.3]	41.1 [23.1, 61.7]	-22.0 [-32.1, -10.5]	-5.3 [-8.6, -1.9]	-0.7 [-1.6, 0.2]
Age 26-64 x Medicaid expansion x Year 3 after base year	10.9 [4.9, 17.3]	44.6 [26.7, 64.9]	-26.7 [-37.5, -14.0]	-5.4 [-9.5, -1.0]	-0.7 [-2.4, 1.0]
Age 26-64 x Medicaid expansion x Year 4 after base year	11.8 [-2.6, 28.3]	44.7 [14.7, 82.5]	-22.9 [-40.0, -0.8]	-3.9 [-9.3, 1.9]	0.6 [-4.4, 5.9]

Notes:
1) The estimates in each column are from a separate regression using observations for the respective payer group/s.
2) Estimates reported here from the log-linear regression model specification.
3) The estimate for each covariate is obtained as 100*[exp(coefficient) - 1].
4) Confidence intervals were obtained based on standard errors clustered at the state level. Estimates in bold indicate significance at $p<0.05$ level.

eFigure 4. Longitudinal rate of bariatric surgery by insurance payer and state Medicaid expansion status

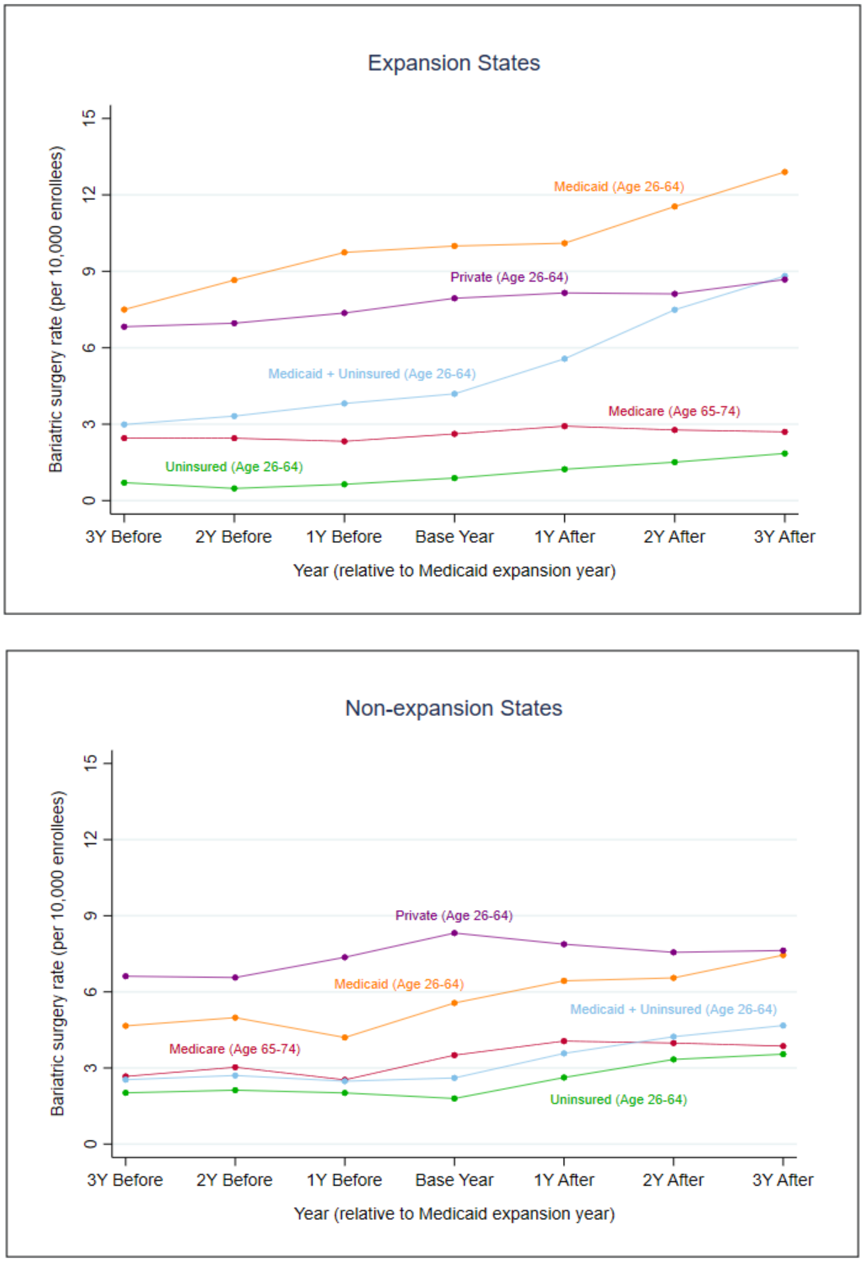

Notes:
1) Observed rate of bariatric surgery by insurance coverage from each state were aggregated for Medicaid expansion and non-expansion states separately. Rate of bariatric surgery was defined as the number of bariatric surgeries for 10,000 census population for each payer cohort each year.
2) For each state, base year is defined as either 2014 (Pennsylvania) or 2013 (other 16 states). Other years are defined relative to the base year for each state. Data for all years (2010-2017) was not available for all the 17 study states. Specifically, we lacked 2010-2011 data for WI and 2017 data for AR and NY. As a result, we have 3-year before and 3-year after base year for all states except WI. The above graphs are reported for the 16 study states other than WI.

eTable 6. Rate and Change in Bariatric Surgery

eTable 6a1. Rate of bariatric surgery by relative year

Insurance payer	3 year before base year	2 year before base year	1 year before base year	Base year (year before expansion)	Year 1 after expansion	Year 2 after expansion	Year 3 after expansion
	Expansion states						
Medicaid & uninsured	3.0	3.3	3.8	4.2	5.6	7.5	8.8
Medicaid	7.5	8.7	9.7	10.0	10.1	11.5	12.9
Uninsured	0.7	0.5	0.6	0.9	1.2	1.5	1.9
Private	6.8	7.0	7.4	7.9	8.2	8.1	8.7
Others	7.5	7.2	6.7	7.5	8.1	7.9	7.7
Age 26-64	49.4	47.4	43.7	48.8	54.9	56.5	57.2
Age 65-74	2.5	2.4	2.3	2.6	2.9	2.8	2.7
All payers	6.4	6.5	6.9	7.6	8.2	8.8	9.5
	Non-expansion states						
Medicaid & uninsured	2.5	2.7	2.5	2.6	3.6	4.2	4.7
Medicaid	4.7	5.0	4.2	5.6	6.4	6.6	7.4
Uninsured	2.0	2.1	2.0	1.8	2.6	3.3	3.5
Private	6.6	6.6	7.4	8.3	7.9	7.6	7.6
Others	8.1	8.6	9.2	8.9	9.6	9.5	9.3
Age 26-64	43.6	44.4	53.3	45.7	47.2	49.6	49.5
Age 65-74	2.7	3.0	2.5	3.5	4.1	4.0	3.9
All payers	6.1	6.1	6.8	7.3	7.5	7.6	7.8

eTable 6a2. Longitudinal change in rate of bariatric surgery: Base year = 100

Insurance payer	3 year before base year	2 year before base year	1 year before base year	Base year (year before expansion)	Year 1 after expansion	Year 2 after expansion	Year 3 after expansion
	Expansion states						
Medicaid & uninsured	71.3	79.2	91.0	100	132.9	178.8	210.4
Medicaid	75.0	86.6	97.5	100	101.1	115.5	129.1
Uninsured	79.5	54.1	72.3	100	139.0	170.4	209.0
Private	85.9	87.7	92.7	100	102.7	102.2	109.3
Others	99.8	95.9	89.0	100	108.5	105.4	102.5
Age 26-64	101.4	97.1	89.5	100	112.5	115.9	117.3
Age 65-74	93.7	93.5	88.9	100	111.6	105.9	103.1
All payers	84.5	86.4	91.6	100	109.1	116.2	126.3
	Non-expansion states						
Medicaid & uninsured	90.8	96.6	84.5	100	123.1	134.4	149.4
Medicaid	83.8	89.6	75.5	100	115.7	117.8	133.9
Uninsured	112.5	118.3	112.2	100	146.0	185.7	197.2
Private	79.5	78.9	88.5	100	94.7	90.9	91.7
Others	94.0	96.2	113.3	100	104.1	108.9	108.3
Age 26-64	95.3	97.0	116.4	100	103.2	108.5	108.2
Age 65-74	76.1	86.5	72.3	100	115.9	113.7	110.2
All payers	91.8	94.1	106.9	100	105.1	109.5	110.8

Notes:
1) Observed rate of bariatric surgery by insurance coverage from each state were aggregated for Medicaid expansion and non-expansion states separately. Rate of bariatric surgery was defined as the number of bariatric surgeries for 10,000 census population for each payer cohort each year.
2) For each state, base year is defined as either 2014 (Pennsylvania) or 2013 (other 16 states). Other years are defined relative to the base year for each state. Data for all years (2010-2017) was not available for all the 17 study states. Specifically, we lacked 2010-2011 data for WI and 2017 data for AR and NY. As a result, we have 3-year before and 3-year after base year for all states except WI. The above graphs are reported for the 16 study states other than WI.

eTable 6b. Change (%) in rate of bariatric surgery associated with Medicaid expansion: Full model estimates

Regression covariate	Medicaid + Uninsured	Medicaid	Uninsured	Private	All payer groups
Age 26-64 (reference: age 65+)	-21.3 [-52.3, 29.9]	56.1 [-26.8, 233.3]	-51.3 [-63.5, -35.1]	120.6 [54.5, 215.1]	107.8 [51.6, 184.7]
Age 26-64 x Medicaid expansion	87.3 [-5.0, 269.4]	109.5 [-9.6, 385.1]	-27.0 [-52.8, 13.0]	34.6 [-11.2, 103.9]	35.8 [-6.1, 96.3]
Year 3 before base year	-23.1 [-36.4, -6.9]	-23.1 [-36.1, -7.6]	-25.4 [-37.9, -10.4]	-25.9 [-39.4, -9.4]	-25.3 [-38.5, -9.3]
Year 2 before base year	-9.3 [-29.0, 15.9]	-9.4 [-28.2, 14.4]	-12.1 [-30.8, 11.7]	-12.7 [-31.1, 10.7]	-11.9 [-30.3, 11.2]
Year 1 before base year	-22.9 [-38.8, -3.0]	-22.9 [-38.8, -3.0]	-22.9 [-38.8, -3.0]	-22.9 [-38.8, -3.0]	-22.9 [-38.8, -3.0]
Year 1 after base year	16.0 [9.3, 23.1]	16.0 [9.3, 23.1]	16.0 [9.3, 23.1]	16.0 [9.3, 23.1]	16.0 [9.3, 23.1]
Year 2 after base year	16.1 [5.5, 27.7]	16.1 [5.5, 27.7]	16.1 [5.5, 27.7]	16.1 [5.5, 27.7]	16.1 [5.5, 27.7]
Year 3 after base year	9.2 [-0.4, 19.7]	9.2 [-0.4, 19.7]	9.2 [-0.4, 19.7]	9.2 [-0.4, 19.7]	9.2 [-0.4, 19.7]
Year 4 after base year	7.6 [1.4, 14.2]	7.6 [1.4, 14.2]	7.6 [1.4, 14.2]	7.6 [1.4, 14.2]	7.6 [1.4, 14.2]
Age 26-64 x Year 3 before base year	17.7 [-25.8, 86.6]	15.5 [-31.9, 96.0]	29.2 [-10.0, 85.5]	10.8 [-8.8, 34.7]	11.7 [-6.5, 33.5]
Age 26-64 x Year 2 before base year	-2.8 [-50.4, 90.4]	-5.2 [-54.7, 98.5]	11.2 [-36.8, 95.8]	-7.0 [-26.6, 17.8]	-4.4 [-24.3, 20.7]
Age 26-64 x Year 1 before base year	11.5 [-38.3, 101.7]	-5.2 [-45.9, 66.1]	20.8 [-30.6, 110.2]	14.8 [-10.7, 47.5]	18.8 [-13.1, 62.5]
Age 26-64 x Year 1 after base year	8.8 [-6.5, 26.6]	-12.2 [-30.8, 11.3]	11.9 [-16.4, 49.8]	-16.3 [-27.1, -4.0]	-10.3 [-20.9, 1.6]
Age 26-64 x Year 2 after base year	28.9 [8.5, 53.2]	-5.2 [-17.7, 9.1]	37.1 [-0.5, 88.8]	-19.2 [-31.7, -4.4]	-9.2 [-22.8, 6.7]
Age 26-64 x Year 3 after base year	54.8 [33.1, 80.0]	17.7 [-3.1, 43.0]	55.4 [13.9, 111.9]	-13.6 [-29.5, 6.0]	-1.6 [-19.2, 19.7]
Age 26-64 x Year 4 after base year	63.9 [32.3, 103.1]	36.1 [8.9, 70.1]	49.1 [-4.9, 133.6]	-14.1 [-32.2, 8.9]	-1.0 [-20.8, 23.8]
Medicaid expansion x Year 3 before base year	23.3 [-15.6, 80.2]	23.4 [-15.3, 79.9]	27.2 [-12.7, 85.3]	28.0 [-12.9, 88.1]	27.0 [-13.3, 85.9]
Medicaid expansion x Year 2 before base year	5.5 [-31.3, 62.1]	5.6 [-30.8, 61.2]	8.9 [-28.9, 66.7]	9.6 [-28.3, 67.5]	8.7 [-28.8, 65.8]
Medicaid expansion x Year 1 before base year	18.1 [-14.4, 63.0]	18.1 [-14.4, 63.0]	18.1 [-14.4, 63.0]	18.1 [-14.4, 63.0]	18.1 [-14.4, 63.0]
Medicaid expansion x Year 1 after base year	-3.9 [-15.0, 8.6]	-3.9 [-15.0, 8.6]	-3.9 [-15.0, 8.6]	-3.9 [-15.0, 8.6]	-3.9 [-15.0, 8.6]
Medicaid expansion x Year 2 after base year	-8.2 [-20.3, 5.8]	-8.2 [-20.3, 5.8]	-8.2 [-20.3, 5.8]	-8.2 [-20.3, 5.8]	-8.2 [-20.3, 5.8]
Medicaid expansion x Year 3 after base year	-3.7 [-16.9, 11.6]	-3.7 [-16.9, 11.6]	-3.7 [-16.9, 11.6]	-3.7 [-16.9, 11.6]	-3.7 [-16.9, 11.6]
Medicaid expansion x Year 4 after base year	-0.9 [-25.4, 31.7]	-2.6 [-25.4, 27.1]	-9.9 [-33.3, 21.7]	-10.8 [-31.5, 16.3]	-8.9 [-29.9, 18.5]
Age 26-64 x Medicaid expansion x Year 3 before base year	-35.2 [-64.2, 17.5]	-31.9 [-66.7, 39.5]	-30.6 [-54.3, 5.3]	-18.7 [-37.6, 6.0]	-19.0 [-36.6, 3.5]

Age 26-64 x Medicaid expansion x Year 2 before base year	-13.3 [-60.9, 92.6]	-2.5 [-60.2, 138.7]	-38.0 [-66.5, 14.7]	-3.7 [-29.1, 30.8]	-4.7 [-28.6, 27.2]
Age 26-64 x Medicaid expansion x Year 1 before base year	-7.9 [-51.2, 73.9]	16.3 [-37.0, 115.0]	-28.5 [-59.6, 26.7]	-10.7 [-32.1, 17.5]	-14.1 [-38.6, 20.0]
Age 26-64 x Medicaid expansion x Year 1 after base year	8.0 [-10.8, 30.7]	5.4 [-18.3, 36.0]	7.4 [-25.5, 54.8]	9.1 [-7.0, 28.0]	8.2 [-6.8, 25.7]
Age 26-64 x Medicaid expansion x Year 2 after base year	29.3 [0.7, 66.0]	20.9 [-3.7, 51.8]	2.3 [-31.1, 51.8]	16.6 [-5.6, 44.0]	19.3 [-1.0, 43.8]
Age 26-64 x Medicaid expansion x Year 3 after base year	30.3 [-0.8, 71.1]	11.6 [-16.3, 48.8]	8.6 [-26.2, 59.8]	18.1 [-7.0, 50.0]	20.8 [-3.0, 50.4]
Age 26-64 x Medicaid expansion x Year 4 after base year	11.1 [-34.2, 87.7]	-10.5 [-41.4, 36.7]	7.9 [-39.4, 92.1]	8.8 [-17.9, 44.2]	7.8 [-20.3, 45.9]

Notes:
1) The estimates in each column are from a separate regression using observations for the respective payer group/s.
2) Estimates reported here from the log-linear regression model specification.
3) The estimate for each covariate is obtained as 100*[exp(coefficient) - 1].
4) Confidence intervals were obtained based on standard errors clustered at the state level. Estimates in bold indicate significance at $p<0.05$ level.

eTable 7. Change by Age and Sex age in bariatric surgery volume and rate

eTable 7a. Change (%) by age in bariatric surgery volume and rate associated with Medicaid expansion among Medicaid covered + Uninsured

Age	Baseline surgery volume / census population (thousands) / surgery rate (#/10,000 population)		Model 1: Change (%) in census population by post-reform year						
	Expansion states	Non-expansion states	3rd year before base year	2nd year before expansion	1 year before base year	Year 1 after expansion	Year 2 after expansion	Year 3 after expansion	Year 4 after expansion
Volume of bariatric surgery									
26-44	5165	2163	-32.8 [-68.2, 42.0]	-11.0 [-63.6, 117.4]	3.1 [-49.5, 110.5]	14.4 [-3.6, 35.8]	**41.3 [5.8, 88.7]**	39.8 [-0.1, 95.7]	19.5 [-30.8, 106.2]
45-54	2473	1344	-46.4 [-67.0, -12.8]	-21.0 [-64.0, 73.2]	-20.3 [-58.8, 54.1]	11.4 [-17.8, 50.9]	**46.7 [18.4, 81.7]**	**52.4 [21.0, 91.9]**	33.2 [-20.3, 122.8]
Census population of Medicaid covered + Uninsured									
26-44	10461	7553	-2.2 [-10.1, 6.3]	-1.4 [-8.8, 6.6]	-0.2 [-3.4, 3.2]	**3.7 [1.6, 5.7]**	**8.2 [3.6, 13.0]**	**8.3 [1.9, 15.2]**	10.2 [-4.5, 27.2]
45-54	7765.0	5534.0	-0.7 [-9.3, 8.8]	-2.7 [-9.8, 5.1]	-0.1 [-3.4, 3.4]	**6.4 [3.9, 8.9]**	**13.0 [7.0, 19.3]**	**14.5 [8.3, 21.2]**	13.9 [-0.4, 30.3]
Rate of bariatric surgery									
26-44	48.8	18.7	-29.8 [-64,9, 40.6]	-10.4 [-60.9, 105.5]	0.4 [-47.5, 92.0]	10.4 [-6.2, 29.8]	30.6 [-0.9, 72.2]	30.1 [-5.9, 79.9]	8.8 [-36.8, 87.2]
45-54	35.4	12.9	-44.4 [-64.2, -13.5]	-19.7 [-61.2, 66.2]	-21.4 [-56.7, 42.5]	4.0 [-22.1, 38.8]	**28.8 [3.0, 61.3]**	**32.6 [3.7, 69.6]**	14.9 [-32.1, 94.4]

Notes:
1) The estimates in each row are from a separate regression using observations for the respective age group. Each regression was based on 264 observations, consisting of 17 states x (up to) 8 years x 2 age groups (26-64 and 65-74). As noted in Table 1, we had 8 years of data for all states, except WI (7 years) and AR and NY (7 years).
2) Estimates reported here from the log-linear regression model specification (Model 1).
3) The estimated change in each of the three outcome measures associated with Medicaid expansion is obtained as 100*[exp(coefficient) - 1] and denotes the percentage change in outcome in the expansion states among those aged 26-64 relative to those aged 65-75 within each state and those aged 26-64 in non-expansion states. The estimates of percent change in each outcome measure in the base year as the reference year. Base year is the year preceding the expansion year, which is 2013 for all states except Pennsylvania (for which 2014 was the base year). The percent change estimates reflect the change associated with Medicaid expansion. See Online Supplement for the model specification details.
4) Confidence intervals were obtained based on standard errors clustered at the state level. Estimates in bold indicate significance at p<0.05 level.
5) Note that the first two columns (baseline levels) give the absolute levels of the three outcome measures: total volume of bariatric surgery, count of census population by payer and rate of bariatric surgery (# surgeries per 10,000 census population by payer). The remaining columns are all in percentage terms, indicating the percentage change in the respective outcome measure associated with Medicaid expansion.

eTable 7b. Change (%) by sex in bariatric surgery volume and rate associated with Medicaid expansion among Medicaid covered + Uninsured

Sex	Baseline surgery volume / census population (thousands) / surgery rate (#/10,000 population)		Model 1: Change (%) in census population by post-reform year						
	Expansion states	Non-expansion states	3rd year before base year	2nd year before expansion	1 year before base year	Year 1 after expansion	Year 2 after expansion	Year 3 after expansion	Year 4 after expansion
Volume of bariatric surgery									
Female	6581	2835	-34.1 [-64.5, 22.5]	-8.9 [-62.3, 120.0]	-0.6 [-50.3, 99.0]	15.9 [-6.7, 44.0]	**48.8 [13.5, 95.1]**	**47.7 [14.1, 91.3]**	20.7 [-31.3, 111.9]
Male	1057	672	-49.6 [-74.2, -1.4]	-30.9 [-67.6, 47.3]	-23.1 [-62.9, 59.5]	6.4 [-21.2, 43.8]	31.8 [-1.4, 76.0]	46.1 [-4.9, 124.6]	33.9 [-29.8, 155.2]
Census population of Medicaid covered + Uninsured									
Female	8970	6482	-0.6 [-8.4, 7.9]	-0.9 [-7.8, 6.4]	0.8 [-2.0, 3.7]	**6.6 [3.9, 9.4]**	**12.8 [7.5, 18.4]**	**14.2 [8.0, 20.6]**	**15.7 [0.9, 32.7]**
Male	9255.0	6605.0	-2.8 [-11.5, 6.7]	-3.1 [-11.0, 5.5]	-1.1 [-4.6, 2.5]	**3.0 [1.1, 4.9]**	**7.6 [2.6, 12.8]**	**7.7 [1.5, 14.3]**	7.8 [-6.3, 24.1]
Rate of bariatric surgery									
Female	74.7	27.3	-32.7 [-62.2, 19.8]	-8.7 [-60.1, 108.9]	-3.2 [-49.3, 85.0]	8.5 [-12.2, 34.0]	31.6 [-0.1, 73.3]	29.7 [-0.7, 69.5]	5.9 [-40.0, 87.0]
Male	12.8	5.3	-44.1 [-69.0, 0.9]	-28.3 [-62.9, 38.6]	-23.5 [-59.3, 43.6]	3.5 [-20.2, 34.2]	21.5 [-5.7, 56.4]	34.9 [-7.5, 96.9]	24.8 [-27.8, 115.7]

Notes:
1) The estimates in each row are from a separate regression using observations for the respective sex group. Each regression was based on 264 observations, consisting of 17 states x (up to) 8 years x 2 age groups (26-64 and 65-74). As noted in Table 1, we had 8 years of data for all states, except WI (7 years) and AR and NY (7 years).
2) Estimates reported here from the log-linear regression model specification (Model 1).
3) The estimated change in each of the three outcome measures associated with Medicaid expansion is obtained as 100*[exp(coefficient) - 1] and denotes the percentage change in outcome in the expansion states among those aged 26-64 relative to those aged 65-75 within each state and those aged 26-64 in non-expansion states. The estimates of percent change in each outcome measure in the base year as the reference year. Base year is the year preceding the expansion year, which is 2013 for all states except Pennsylvania (for which 2014 was the base year). The percent change estimates reflect the change associated with Medicaid expansion. See Online Supplement for the model specification details.
4) Confidence intervals were obtained based on standard errors clustered at the state level. Estimates in bold indicate significance at p<0.05 level.
5) Note that the first two columns (baseline levels) give the absolute levels of the three outcome measures: total volume of bariatric surgery, count of census population by payer and rate of bariatric surgery (# surgeries per 10,000 census population by payer). The remaining columns are all in percentage terms, indicating the percentage change in the respective outcome measure associated with Medicaid expansion.

eTable 8. Linear and Poisson Models of Change

eTable 8a. Linear model of the change in volume of bariatric surgeries associated with Medicaid expansion: Overall and by payer

Outcome = # bariatric surgeries (in hundreds)

Regression covariates	Medicaid + Uninsured	Medicaid	Uninsured	Private	All payer groups
Age 26-64 (reference: age 65+)	2.80 [0.81, 4.80]	-1.91 [-5.46, 1.65]	-0.61 [-0.96, -0.26]	43.14 [5.87, 80.40]	60.14 [17.21, 103.08]
Age 26-64 x Medicaid expansion	5.93 [-1.00, 12.86]	9.19 [2.06, 16.33]	-1.54 [-2.83, -0.25]	10.06 [-41.02, 61.13]	12.96 [-47.39, 73.30]
Year 3 before base year	-2.13 [-4.10, -0.16]	-1.90 [-3.78, -0.02]	-2.06 [-4.06, -0.05]	-3.39 [-7.66, 0.88]	-4.00 [-9.48, 1.47]
Year 2 before base year	-1.68 [-3.72, 0.37]	-1.45 [-3.40, 0.51]	-1.61 [-3.69, 0.48]	-2.94 [-7.25, 1.38]	-3.55 [-9.06, 1.96]
Year 1 before base year	-1.86 [-3.76, 0.05]	-1.86 [-3.76, 0.05]	-1.86 [-3.76, 0.05]	-1.86 [-3.76, 0.05]	-1.86 [-3.76, 0.05]
Year 1 after base year	1.08 [0.46, 1.70]	1.08 [0.46, 1.70]	1.08 [0.46, 1.70]	1.08 [0.46, 1.70]	1.08 [0.46, 1.70]
Year 2 after base year	1.17 [0.67, 1.68]	1.17 [0.67, 1.68]	1.17 [0.67, 1.68]	1.17 [0.67, 1.68]	1.17 [0.67, 1.68]
Year 3 after base year	1.28 [0.57, 2.00]	1.28 [0.57, 2.00]	1.28 [0.57, 2.00]	1.28 [0.57, 2.00]	1.28 [0.57, 2.00]
Year 4 after base year	1.56 [0.57, 2.56]	1.56 [0.57, 2.56]	1.56 [0.57, 2.56]	1.56 [0.57, 2.56]	1.56 [0.57, 2.56]
Age 26-64 x Year 3 before base year	2.03 [-2.64, 6.70]	0.95 [-2.29, 4.20]	2.71 [-0.71, 6.13]	-6.95 [-17.43, 3.54]	-6.99 [-17.39, 3.41]
Age 26-64 x Year 2 before base year	2.64 [-3.80, 9.08]	1.02 [-2.77, 4.82]	2.80 [-1.87, 7.46]	-8.08 [-19.57, 3.41]	-6.67 [-17.36, 4.03]
Age 26-64 x Year 1 before base year	2.13 [-2.64, 6.90]	1.21 [-1.10, 3.52]	2.78 [-1.52, 7.07]	-3.76 [-6.36, -1.15]	-1.15 [-6.79, 4.49]
Age 26-64 x Year 1 after base year	0.64 [-0.74, 2.01]	-0.61 [-2.18, 0.96]	0.17 [-0.26, 0.60]	-1.59 [-8.55, 5.38]	1.08 [-7.09, 9.25]
Age 26-64 x Year 2 after base year	1.67 [-0.64, 3.98]	-0.34 [-1.53, 0.86]	0.83 [-0.45, 2.11]	-2.53 [-15.36, 10.31]	1.31 [-13.64, 16.25]
Age 26-64 x Year 3 after base year	2.40 [-0.27, 5.07]	0.16 [-1.44, 1.76]	0.96 [-0.47, 2.39]	-0.27 [-11.42, 10.88]	4.67 [-9.42, 18.77]
Age 26-64 x Year 4 after base year	2.88 [-0.95, 6.70]	0.42 [-0.90, 1.73]	0.90 [-1.61, 3.41]	-1.39 [-17.25, 14.47]	4.92 [-15.49, 25.33]
Medicaid expansion x Year 3 before base year	1.91 [-0.40, 4.22]	1.68 [-0.56, 3.91]	1.84 [-0.51, 4.18]	3.17 [-1.27, 7.61]	3.78 [-1.83, 9.39]
Medicaid expansion x Year 2 before base year	1.61 [-0.90, 4.11]	1.38 [-1.05, 3.81]	1.54 [-1.00, 4.07]	2.87 [-1.68, 7.42]	3.48 [-2.21, 9.18]
Medicaid expansion x Year 1 before base year	1.59 [-0.46, 3.63]	1.59 [-0.46, 3.63]	1.59 [-0.46, 3.63]	1.59 [-0.46, 3.63]	1.59 [-0.46, 3.63]
Medicaid expansion x Year 1 after base year	-0.65 [-1.31, 0.01]	-0.65 [-1.31, 0.01]	-0.65 [-1.31, 0.01]	-0.65 [-1.31, 0.01]	-0.65 [-1.31, 0.01]
Medicaid expansion x Year 2 after base year	-0.72 [-1.36, -0.07]	-0.72 [-1.36, -0.07]	-0.72 [-1.36, -0.07]	-0.72 [-1.36, -0.07]	-0.72 [-1.36, -0.07]

Medicaid expansion x Year 3 after base year	-0.74 [-1.53, 0.05]	-0.74 [-1.53, 0.05]	-0.74 [-1.53, 0.05]	-0.74 [-1.53, 0.05]	-0.74 [-1.53, 0.05]
Medicaid expansion x Year 4 after base year	0.82 [-2.54, 4.18]	0.80 [-2.39, 4.00]	-0.90 [-2.22, 0.41]	-1.11 [-8.00, 5.79]	0.98 [-9.85, 11.80]
Age 26-64 x Medicaid expansion x Year 3 before base year	-5.84 [-11.76, 0.07]	-4.81 [-9.83, 0.22]	-2.45 [-5.94, 1.05]	4.65 [-9.30, 18.60]	0.41 [-16.03, 16.84]
Age 26-64 x Medicaid expansion x Year 2 before base year	-5.64 [-12.94, 1.66]	-3.58 [-8.66, 1.50]	-3.17 [-7.96, 1.63]	8.65 [-11.18, 28.49]	3.75 [-17.27, 24.77]
Age 26-64 x Medicaid expansion x Year 1 before base year	-3.08 [-8.06, 1.90]	-1.85 [-4.51, 0.80]	-2.81 [-7.13, 1.51]	3.07 [-5.00, 11.15]	-1.59 [-12.08, 8.90]
Age 26-64 x Medicaid expansion x Year 1 after base year	1.97 [-0.18, 4.12]	3.15 [0.90, 5.40]	-0.53 [-1.07, -0.00]	3.68 [-3.61, 10.97]	5.18 [-3.76, 14.13]
Age 26-64 x Medicaid expansion x Year 2 after base year	5.25 [0.63, 9.87]	7.31 [3.03, 11.60]	-1.34 [-2.77, 0.09]	3.69 [-10.05, 17.44]	8.38 [-6.90, 23.65]
Age 26-64 x Medicaid expansion x Year 3 after base year	7.38 [1.16, 13.60]	9.64 [3.55, 15.72]	-1.51 [-3.12, 0.09]	4.77 [-7.34, 16.88]	11.11 [-3.73, 25.96]
Age 26-64 x Medicaid expansion x Year 4 after base year	5.28 [-10.68, 21.25]	7.83 [-7.42, 23.07]	-1.68 [-4.50, 1.14]	5.06 [-16.18, 26.30]	7.57 [-31.40, 46.54]

Notes:
1) The estimates in each column are from a separate regression using observations for the respective payer group/s.
2) Estimates reported here from the linear regression model specification.
3) The estimate for each covariate denotes count of bariatric surgeries (in hundreds).
4) Confidence intervals were obtained based on standard errors clustered at the state level. Estimates in bold indicate significance at $p<0.05$ level.

eTable 8b. Poisson model of the change in volume of bariatric surgeries associated with Medicaid expansion: Overall and by payer

Regression covariates	Medicaid + Uninsured	Medicaid	Uninsured	Private	All payer groups
Age 26-64 (reference: age 65+)	61.5 [27.4, 104.8]	-20.9 [-55.4, 40.4]	-17.5 [-32.1, 0.2]	873.7 [556.2, 1344.7]	1227.8 [892.8, 1675.9]
Age 26-64 x Medicaid expansion	94.9 [16.7, 225.6]	244.5 [60.3, 640.2]	-48.6 [-64.9, -24.6]	41.1 [-9.7, 120.4]	43.7 [0.7, 105.1]
Year 3 before base year	-33.9 [-47.4, -16.9]	-32.5 [-46.4, -15.0]	-35.6 [-49.2, -18.3]	-34.9 [-49.0, -17.0]	-34.6 [-48.6, -16.8]
Year 2 before base year	-22.6 [-39.2, -1.5]	-21.0 [-38.1, 1.0]	-24.6 [-40.9, -3.9]	-23.8 [-40.5, -2.5]	-23.5 [-40.1, -2.2]
Year 1 before base year	-30.5 [-42.8, -15.6]	-30.5 [-42.8, -15.6]	-30.5 [-42.8, -15.6]	-30.5 [-42.8, -15.6]	-30.5 [-42.8, -15.6]
Year 1 after base year	21.4 [17.5, 25.4]	21.4 [17.5, 25.4]	21.4 [17.5, 25.4]	21.4 [17.5, 25.4]	21.4 [17.5, 25.4]
Year 2 after base year	25.6 [16.4, 35.5]	25.6 [16.4, 35.5]	25.6 [16.4, 35.5]	25.6 [16.4, 35.5]	25.6 [16.4, 35.5]
Year 3 after base year	25.2 [19.2, 31.6]	25.2 [19.2, 31.6]	25.2 [19.2, 31.6]	25.2 [19.2, 31.6]	25.2 [19.2, 31.6]
Year 4 after base year	29.4 [22.8, 36.3]	29.4 [22.8, 36.3]	29.4 [22.8, 36.3]	29.4 [22.8, 36.3]	29.4 [22.8, 36.3]
Age 26-64 x Year 3 before base year	36.6 [-16.3, 122.9]	0.4 [-41.2, 71.7]	71.3 [18.3, 148.0]	19.7 [-3.5, 48.4]	23.3 [-1.8, 54.9]
Age 26-64 x Year 2 before base year	28.0 [-31.4, 138.8]	-2.5 [-48.7, 85.6]	57.2 [-5.6, 161.9]	1.6 [-17.6, 25.2]	7.4 [-15.0, 35.5]
Age 26-64 x Year 1 before base year	38.5 [-13.4, 121.4]	15.5 [-17.7, 62.1]	60.5 [-2.8, 165.0]	26.8 [3.6, 55.2]	33.4 [1.4, 75.6]
Age 26-64 x Year 1 after base year	0.0 [-8.8, 9.7]	-4.4 [-19.6, 13.7]	4.3 [-2.1, 11.2]	-17.6 [-24.7, -9.8]	-14.4 [-21.8, -6.3]
Age 26-64 x Year 2 after base year	6.3 [-7.1, 21.6]	-0.9 [-14.0, 14.2]	13.2 [-4.0, 33.4]	-19.5 [-29.3, -8.4]	-15.2 [-24.8, -4.3]
Age 26-64 x Year 3 after base year	15.5 [2.0, 30.8]	13.2 [-2.3, 31.2]	17.7 [-1.1, 40.2]	-17.0 [-29.0, -2.9]	-12.0 [-24.5, 2.6]
Age 26-64 x Year 4 after base year	20.7 [2.3, 42.3]	21.4 [4.5, 41.1]	19.9 [-12.7, 64.8]	-21.9 [-35.3, -5.7]	-14.8 [-29.4, 2.7]
Medicaid expansion x Year 3 before base year	22.7 [-14.0, 75.1]	20.1 [-15.9, 71.5]	26.0 [-12.2, 80.7]	24.7 [-13.5, 79.6]	24.1 [-13.7, 78.4]
Medicaid expansion x Year 2 before base year	8.5 [-26.2, 59.5]	6.2 [-28.0, 56.5]	11.3 [-24.4, 63.8]	10.2 [-25.3, 62.6]	9.7 [-25.6, 61.7]
Medicaid expansion x Year 1 before base year	21.9 [-6.9, 59.6]	21.9 [-6.9, 59.6]	21.9 [-6.9, 59.6]	21.9 [-6.9, 59.6]	21.9 [-6.9, 59.6]
Medicaid expansion x Year 1 after base year	-3.7 [-12.0, 5.4]	-3.7 [-12.0, 5.4]	-3.7 [-12.0, 5.4]	-3.7 [-12.0, 5.4]	-3.7 [-12.0, 5.4]
Medicaid expansion x Year 2 after base year	-7.8 [-19.0, 4.8]	-7.8 [-19.0, 4.8]	-7.8 [-19.0, 4.8]	-7.8 [-19.0, 4.8]	-7.8 [-19.0, 4.8]
Medicaid expansion x Year 3 after base year	-6.8 [-16.0, 3.4]	-6.8 [-16.0, 3.4]	-6.8 [-16.0, 3.4]	-6.8 [-16.0, 3.4]	-6.8 [-16.0, 3.4]
Medicaid expansion x Year 4 after base year	13.7 [-16.6, 55.0]	16.6 [-15.4, 60.8]	-11.4 [-26.4, 6.7]	-5.3 [-19.8, 11.8]	-0.7 [-17.9, 20.0]
Age 26-64 x Medicaid expansion x Year 3 before base year	-37.8 [-65.3, 11.4]	-17.6 [-57.2, 58.7]	-42.4 [-62.2, -12.2]	-11.5 [-31.4, 14.2]	-16.5 [-36.2, 9.2]
Age 26-64 x Medicaid expansion x Year 2 before base year	-26.5 [-63.9, 49.9]	1.0 [-52.0, 112.7]	-58.1 [-76.1, -26.4]	2.3 [-20.2, 31.2]	-4.7 [-27.3, 24.7]
Age 26-64 x Medicaid expansion x Year 1 before base year	-22.5 [-53.0, 27.9]	-4.3 [-35.1, 41.2]	-45.7 [-67.9, -7.9]	-14.0 [-30.9, 7.0]	-19.2 [-39.6, 8.0]

Age 26-64 x Medicaid expansion x Year 1 after base year	7.8 [-4.8, 22.2]	15.7 [-4.8, 40.6]	-13.2 [-29.5, 7.0]	10.0 [-1.7, 23.0]	9.8 [-1.7, 22.8]
Age 26-64 x Medicaid expansion x Year 2 after base year	34.1 [8.1, 66.4]	52.8 [20.4, 93.9]	-23.9 [-44.8, 4.9]	15.4 [-2.6, 36.7]	19.9 [3.1, 39.3]
Age 26-64 x Medicaid expansion x Year 3 after base year	41.6 [11.8, 79.3]	54.0 [16.4, 103.7]	-20.0 [-43.0, 12.3]	19.5 [-1.2, 44.6]	24.4 [4.2, 48.7]
Age 26-64 x Medicaid expansion x Year 4 after base year	19.6 [-28.4, 99.9]	25.9 [-28.1, 120.5]	-25.5 [-59.9, 38.4]	19.0 [-5.6, 50.0]	17.9 [-8.6, 52.1]

Notes:
1) The estimates in each column are from a separate regression using observations for the respective payer group/s.
2) Estimates reported here from the Poisson regression model specification.
3) The estimate for each covariate is obtained as 100*[exp(coefficient) - 1].
4) Confidence intervals were obtained based on standard errors clustered at the state level. Estimates in bold indicate significance at $p<0.05$ level.

eTable 9. Bariatric Surgery by Type

As noted previously (eTable 2a, 2b), two procedures - laparoscopic Roux-en-Y Gastric Bypass (RYGB) gastric bypass and sleeve gastrectomy (SG) procedures – account for majority of bariatric surgeries. Over time the share of SG has been rising. Following tables quantify these trends separately by expansion and non-expansion states.

eTable 9a1. Number of bariatric procedures by type

Procedure type	2010	2011	2012	2013	2014	2015	2016
	Expansion states						
Total	41,047	41,714	43,635	48,382	53,496	56,783	61,145
RYGB	23,866	22,086	20,489	17,962	16,355	15,243	15,474
VSG	0	3,438	18,070	27,413	34,995	39,648	44,436
Other	17,181	16,190	5,076	3,007	2,146	1,892	1,235
	Non-expansion states						
Total	23,645	24,300	26,819	29,523	31,309	32,121	33,255
RYGB	13,994	12,630	11,990	10,877	10,194	9,515	8,298
VSG	0	2,040	12,107	17,014	19,686	21,029	24,041
Other	9,651	9,630	2,722	1,632	1,429	1,577	916

eTable 9a2. Share of total annual volume

Procedure type	2010	2011	2012	2013	2014	2015	2016
	Expansion states						
RYGB	58.1%	53.0%	47.0%	37.1%	30.6%	26.8%	25.3%
VSG	0.0%	8.2%	41.4%	56.7%	65.4%	69.8%	72.7%
Other	41.9%	38.8%	11.6%	6.2%	4.0%	3.3%	2.0%
	Non-expansion states						
RYGB	59.2%	52.0%	44.7%	36.8%	32.6%	29.6%	25.0%
VSG	0.0%	8.4%	45.1%	57.6%	62.9%	65.5%	72.3%
Other	40.8%	39.6%	10.2%	5.5%	4.6%	4.9%	2.8%

eTable 9b. Change in volume of bariatric surgeries associated with Medicaid expansion: Revised surgery identification

Regression covariates	Medicaid + Uninsured	Medicaid	Uninsured	Private	All payer groups
Age 26-64 (reference: age 65+)	62.8 [10.9, 139.0]	-25.8 [-66.3, 63.4]	-25.7 [-48.9, 8.1]	886.3 [488.1, 1554.2]	1314.2 [857.6, 1988.4]
Age 26-64 x Medicaid expansion	88.8 [7.0, 233.4]	239.3 [34.5, 755.7]	-45.9 [-68.5, -7.4]	53.0 [-14.6, 174.4]	47.4 [-6.8, 133.2]
Year 3 before base year	-67.1 [-75.4, -55.8]	-66.2 [-74.8, -54.6]	-68.7 [-77.1, -57.4]	-67.8 [-76.0, -56.8]	-67.7 [-75.8, -56.8]
Year 2 before base year	-55.2 [-65.3, -42.1]	-54.0 [-64.6, -40.1]	-57.5 [-67.6, -44.3]	-56.2 [-65.4, -44.6]	-56.0 [-65.3, -44.3]
Year 1 before base year	-38.0 [-51.7, -20.4]	-38.0 [-51.7, -20.4]	-38.0 [-51.7, -20.4]	-38.0 [-51.7, -20.4]	-38.0 [-51.7, -20.4]
Year 1 after base year	26.2 [17.1, 36.0]	26.2 [17.1, 36.0]	26.2 [17.1, 36.0]	26.2 [17.1, 36.0]	26.2 [17.1, 36.0]
Year 2 after base year	33.6 [19.6, 49.2]	33.6 [19.6, 49.2]	33.6 [19.6, 49.2]	33.6 [19.6, 49.2]	33.6 [19.6, 49.2]
Year 3 after base year	32.9 [18.2, 49.5]	32.9 [18.2, 49.5]	32.9 [18.2, 49.5]	32.9 [18.2, 49.5]	32.9 [18.2, 49.5]
Year 4 after base year	36.8 [25.6, 49.0]	36.8 [25.6, 49.0]	36.8 [25.6, 49.0]	36.8 [25.6, 49.0]	36.8 [25.6, 49.0]
Age 26-64 x Year 3 before base year	31.1 [-3.9, 79.1]	78.7 [-10.2, 255.3]	2.1 [-14.9, 22.5]	60.4 [32.8, 93.9]	55.9 [28.2, 89.5]
Age 26-64 x Year 2 before base year	8.4 [-28.0, 63.4]	34.6 [-37.3, 189.3]	-1.5 [-25.0, 29.5]	16.4 [-3.1, 39.7]	20.2 [5.6, 36.9]
Age 26-64 x Year 1 before base year	29.5 [-34.1, 154.6]	7.5 [-45.3, 111.1]	41.0 [-23.9, 160.9]	36.5 [6.0, 75.7]	39.8 [1.2, 92.9]
Age 26-64 x Year 1 after base year	-6.4 [-21.4, 11.5]	-13.1 [-31.7, 10.6]	-10.6 [-40.4, 34.1]	-17.7 [-28.7, -5.0]	-15.5 [-25.7, -3.9]
Age 26-64 x Year 2 after base year	-2.3 [-16.5, 14.4]	-9.6 [-23.2, 6.5]	-6.0 [-34.0, 34.0]	-21.3 [-33.2, -7.4]	-18.4 [-29.6, -5.4]
Age 26-64 x Year 3 after base year	11.8 [0.8, 24.0]	13.3 [-6.9, 37.9]	-1.0 [-28.4, 36.8]	-17.5 [-32.6, 1.1]	-14.0 [-28.4, 3.3]
Age 26-64 x Year 4 after base year	17.7 [1.7, 36.2]	26.4 [1.2, 57.9]	-6.5 [-42.1, 51.1]	-20.5 [-37.9, 1.9]	-15.6 [-32.0, 4.8]
Medicaid expansion x Year 3 before base year	16.0 [-34.8, 106.5]	12.9 [-36.6, 100.9]	22.3 [-31.9, 119.4]	18.6 [-33.3, 111.1]	18.2 [-33.5, 109.9]
Medicaid expansion x Year 2 before base year	7.7 [-38.1, 87.5]	4.8 [-40.0, 83.0]	13.5 [-35.3, 98.9]	10.1 [-36.2, 89.9]	9.7 [-36.4, 89.2]
Medicaid expansion x Year 1 before base year	21.7 [-17.4, 79.4]	21.7 [-17.4, 79.4]	21.7 [-17.4, 79.4]	21.7 [-17.4, 79.4]	21.7 [-17.4, 79.4]
Medicaid expansion x Year 1 after base year	-2.2 [-15.8, 13.6]	-2.2 [-15.8, 13.6]	-2.2 [-15.8, 13.6]	-2.2 [-15.8, 13.6]	-2.2 [-15.8, 13.6]
Medicaid expansion x Year 2 after base year	-4.4 [-19.6, 13.6]	-4.4 [-19.6, 13.6]	-4.4 [-19.6, 13.6]	-4.4 [-19.6, 13.6]	-4.4 [-19.6, 13.6]
Medicaid expansion x Year 3 after base year	0.0 [-17.4, 21.0]	0.0 [-17.4, 21.0]	0.0 [-17.4, 21.0]	0.0 [-17.4, 21.0]	0.0 [-17.4, 21.0]
Medicaid expansion x Year 4 after base year	-2.0 [-34.2, 45.8]	-0.4 [-32.8, 47.8]	-13.6 [-44.3, 34.0]	-11.3 [-39.7, 30.4]	-9.8 [-38.6, 32.6]
Age 26-64 x Medicaid expansion x Year 3 before base year	-27.3 [-59.6, 30.8]	-44.8 [-77.3, 34.4]	-11.9 [-39.1, 27.4]	-11.3 [-34.3, 19.8]	-12.6 [-35.7, 18.9]
Age 26-64 x Medicaid expansion x Year 2 before base year	-5.4 [-52.6, 89.0]	-17.7 [-69.2, 119.9]	-27.5 [-52.0, 9.7]	1.1 [-28.8, 43.7]	-1.7 [-28.9, 35.8]

Age 26-64 x Medicaid expansion x Year 1 before base year	-12.4 [-58.2, 83.5]	8.5 [-48.9, 130.3]	-31.7 [-63.8, 28.9]	-13.4 [-35.6, 16.6]	-17.0 [-42.3, 19.4]
Age 26-64 x Medicaid expansion x Year 1 after base year	12.0 [-9.9, 39.2]	27.1 [-3.6, 67.6]	0.7 [-36.6, 59.9]	5.9 [-11.0, 26.1]	7.1 [-8.9, 25.8]
Age 26-64 x Medicaid expansion x Year 2 after base year	39.2 [6.3, 82.3]	67.5 [24.7, 125.1]	-18.3 [-48.5, 29.6]	8.3 [-12.1, 33.5]	15.8 [-3.9, 39.5]
Age 26-64 x Medicaid expansion x Year 3 after base year	40.3 [6.2, 85.3]	53.7 [7.7, 119.4]	-17.7 [-47.0, 27.7]	7.8 [-15.1, 37.0]	15.8 [-7.1, 44.2]
Age 26-64 x Medicaid expansion x Year 4 after base year	19.4 [-29.3, 101.7]	24.7 [-30.0, 122.4]	-9.8 [-53.5, 74.7]	-2.5 [-30.9, 37.6]	-0.3 [-31.8, 45.8]

Notes:
1) Only the volume of the two main two procedures - laparoscopic Roux-en-Y Gastric Bypass (RYGB) gastric bypass and sleeve gastrectomy (SG) – were used to measure surgery volume.
2) The estimates in each column are from a separate regression using observations for the respective payer group/s.
3) Estimates reported here from the log-linear regression model specification.
4) The estimate for each covariate is obtained as 100*[exp(coefficient) - 1].
5) Confidence intervals were obtained based on standard errors clustered at the state level. Estimates in bold indicate significance at $p<0.05$ level.
